# PARS, low-cost portable rehabilitation system for upper arm

**DOI:** 10.1016/j.ohx.2022.e00299

**Published:** 2022-03-23

**Authors:** Mertcan Koçak, Erkin Gezgin

**Affiliations:** Department of Mechatronics Engineering, İzmir Katip Çelebi University, Turkey

**Keywords:** Robotic rehabilitation, Interaction control, Upper arm rehabilitation, Rehabilitation gaming

## Abstract

This study introduces a compact low-cost single degree of freedom end-effector type upper arm rehabilitation system (PARS) along with its hardware and software elements. Proposed system is also suitable to be used in conjunction with a gaming environment. Throughout the study structural setup of the system was explained in detail along with its electronics, control system and gaming software. Introduced virtual gaming interface supports various game levels with different difficulties generated via interaction type control algorithms. Having simple structural design constructed by using basic available components, proposed system can be easily manufactured and utilized in physical rehabilitation procedures by using supplied open source codes. Introduced systems compactness and user friendly interface also allow its usage for individual home therapies for remote rehabilitation treatment procedures.

## Graphical abstract

**Figure:**
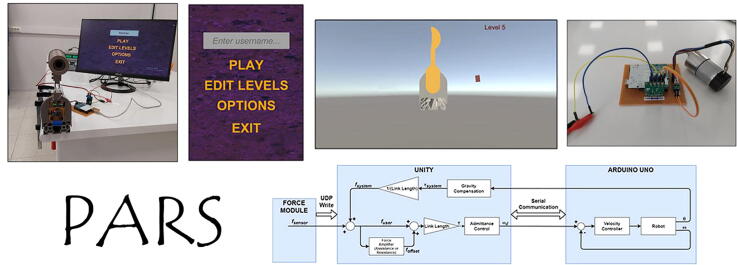

## Specifications table

**Table:** 

Hardware name	PARS (Portable Arm Rehabilitation System)
Subject area	• Engineering • Medical (rehabilitation robotics) • Educational tools and open source alternatives to existing infrastructure
Hardware type	• Electrical engineering and computer science • Robotics • Mechanism and machine science
Open source license	CC BY 4.0
Cost of hardware	170.12 $
Source file repository	https://doi.org/10.17632/wrx22bn64h.2

## Hardware in context

Throughout the life, various reasons may lead people to have temporary or permanent disabilities. Thus without any limitations all human beings are the candidates of such incidents. World Health Organization (WHO) estimates that over one billion people, nearly 15% of global population, live with at least one kind of disability [1]. In foreseeable future this rate is expected to be increased due to chronic health conditions as most of these disabled individuals live in countries with low to middle incomes. Amongst these disabilities, neurological disorders affect the largest number of people, who need intensive physical rehabilitation treatments to improve quality of their lives. Stroke can be seen as one of the main causes of these neurological disorders, where it was reclassified as neurological disease by WHO, leaving its former classification as cardiovascular disease [2].

Functional recovery of the patients in these neurological diseases via plasticity formation is still possible with intensive, repetitive and most importantly immediate rehabilitation treatments after the incidents [3]. In light of this, physical rehabilitation procedures should be utilized in systematic ways that are mainly generalized as passive and active treatment methodologies [4]. In passive treatments repetitive desired motion activities of the patients are actuated by an external source for plasticity formation. This early stage plays a vital role in patient recovery. In active treatments though, patients should provide some level of muscle activities to perform desired motions, which is only possible by gaining the ability to send neurological signals to the affected extremity after plasticity formation. Due to low muscle strength in early stages of active treatments, patients require some amount of assistance with respect to the level of their health condition. Later stages of the active treatments aim to let patients to regain some muscle strength by inducing resistances during the desired motion execution [5]. In classical rehabilitation treatments, these procedures are being applied mostly by trained medical personnel manually. Thus increased rehabilitation demands in conjunction with rapid growing world population raise the need for more healthcare personnel and medical infrastructure [6]. Unfortunately considering the countries with low to middle incomes, these demands cannot be easily met [7].

In order to address this problem for the future, robotic rehabilitation started to gain popularity having some advantages over classical rehabilitation. In this relatively new field, passive and active rehabilitation procedures are utilized by modeling interaction control strategies that are embedded into the robotic systems. Due to the fact that mechanical interaction with objects is very essential for robots that work in collaboration with the humans, new approaches are being proposed throughout the huge amount of robotic applications [8], [9], [10] for efficient human-robot interaction, and researches in rehabilitation robotics started to benefit strongly from these [11], [12]. In order to improve motor functions of the post stroke patients, different types of intelligent controllers were also proposed for various devices [13], [14], [15]. These types mostly include indirect force control strategies generally labeled as impedance and admittance control that create dynamic behavior between applied force/torque and desired motion. In admittance control, motion controllers are used as an inner loop fed by a force controlled external loop. Admittance control mainly focuses on force tracking, preferred in non-backdrivable systems and utilized in many rehabilitation applications throughout the literature for various cases [16], [17], [18].

Although proposed methodologies and robotic rehabilitation treatments offer new opportunities for the future, in order to remedy for increased healthcare personnel and medical infrastructure needs properly, designed systems should be as available as possible for the majority of the target patient population in terms of costs, usability, and portability. Despite many robotic rehabilitation systems that targets different body extremities [19], [20], [21], [22], [23] exist, their designs are generally complex in structure that lead increase in overall costs as well as control algorithm complexity. Thus various researches aim to decrease overall costs and bulkiness of the proposed systems in order to have an accessible system for the majority [24], [25], [26]. Moreover, virtual games are also started to be combined with the robotic rehabilitation treatments to increase patient motivations during performing given therapy tasks [27], [28], [29], [30], [31], [32], [33].

In light of mentioned literature and previous preliminary work of the authors [34], this paper tries to propose a robust, low-cost rehabilitation robotic system that can easily be adapted to various rehabilitation treatment scenarios. In this version of the proposed hardware overall costs were dramatically reduced and improvements on the hardware/software of the system increased system reproducibility.

## Hardware description

**Fig. 1:**
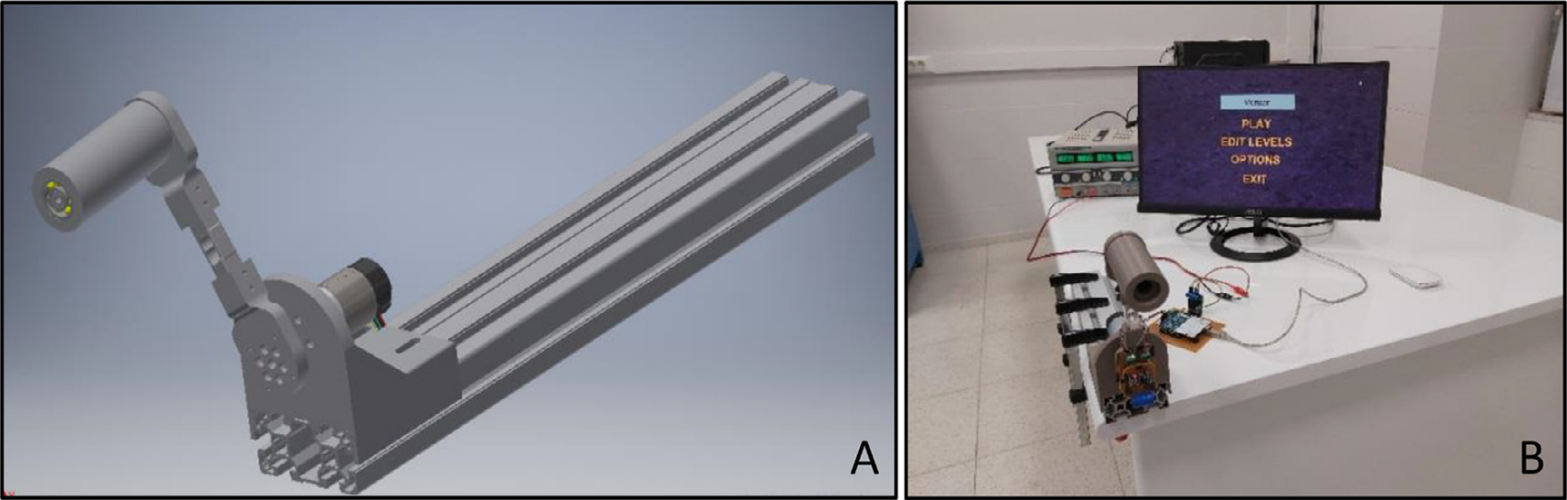
PARS (A) CAD drawing (B) assembled system.

PARS (Portable Arm Rehabilitation System) (Fig. 1) is a low-cost, simple, mobile, and compact single degree of freedom end-effector type upper arm rehabilitation system that helps to execute various upper arm movements such as forearm flexion/extension including supportive shoulder movements. Also, it can be treated as a special hardware setup, on which different type of interaction control algorithms can be utilized. Basically overall system consists of single brushed DC motor equipped with an encoder that actuates the system in a constrained circular pattern by the help of position/velocity feedback, and a single simple load cell that extracts information of user force intention.

Throughout the design of PARS, basic low-cost and available components were utilized so that any person can construct the system and use it without having any advance technical experience. PARS should be treated as an overall system with hardware and software blended together in order to be utilized in rehabilitation field.

In order to be operational, proposed system should be attached to a stable frame. PARS was designed to be built on a 40x80 aluminum extrusion profile base that can be carried to various locations easily. “Motor Holder” is the main component that is fixed to this base. Being systems actuator, DC motor stays fixed on motor holder and it is also used as the assembly housing during load cell calibration procedure. User handle equipped with a load cell can be treated as the force transferring link. Two assembly parts of the link serves for different purposes as the attachment point of the rotary actuator in one end, and user handle with passive rotational degree of freedom on the other end. These two ends are connected together via load cell itself in order to sense load difference between them. Eventually it works as a force feedback tool during user (patient or subject that uses the hardware) interaction. Since load cell itself is located in rotating parts, risk of cable entanglement arises. This problem was solved in authors’ previous work [34] by using a battery powered microcontroller that reads load cell value and transmits extracted data wirelessly. Interpreted data is transferred to the gaming software wirelessly via User Datagram Protocol (UDP). Rehabilitation game and the logic behind the outer loop control were developed in Unity3D [35].

Proposed system is an electro-mechanical system together with a gaming interface. In order to achieve seamless operation, electronic and mechanical components in the system need to be working together properly. Also as the treatment procedure relies on an interaction control strategy, they should be blended each other by an efficient control procedure. Due to the fact that load cell is the main feedback device that measures interaction between the user and the robot, admittance control was chosen as the main interaction control type that creates dynamic behavior between applied force/torque and motion [34].

**Fig. 2:**
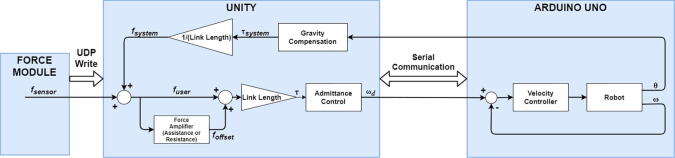
Control and communication block diagram.

In Fig. 2, *f_offset_* represents predefined offset force, which is related with the assistance or resistance in rehabilitation procedures. In the control diagram, admittance control block accepts required torque values and determines desired rotation velocity of application shaft *ω_d_*. This value is sent to inner velocity control loop as the reference value, where the communication between Unity and Arduino Uno is handled via serial communication. Force amplifier block creates offset force (*f_offset_*) utilizing Equation (1), if it is assistive.

(1) $$f_{\mathrm{offset}}=\left\{\begin{array}{c} f_{\mathrm{user}},f_{\mathrm{user}}<f_{\mathrm{saturation}}\\ f_{\mathrm{saturation}},f_{\mathrm{user}}\geq f_{\mathrm{saturation}} \end{array}\right)$$

If the procedure is resistive, then offset force (*f_offset_*) is determined by Equation (2).

(2) $$f_{\mathrm{offset}}=\left\{\begin{array}{c} {-f}_{\mathrm{user}},f_{\mathrm{user}}<f_{\mathrm{saturation}}\\ {-f}_{\mathrm{saturation}},f_{\mathrm{user}}\geq f_{\mathrm{saturation}} \end{array}\right)$$

Offset force works collaboratively or against user that increase or decrease efforts by changing required torque that is applied to the admittance control block. Together with virtual dynamics of admittance control, it strongly affects tendency of rehabilitation procedure. However, when no intention comes from the user (*f_user_* = 0), offset force should be zero as well, since it must not affect required torque that might be possible with modeled equations. In resistive case, movement does not start until *f_saturation_* value is reached by the user, and this affects user effort dramatically.

Modifying given open source design files, different type of actuators and/or sensors can be utilized in the system, so that a new balance between cost and component quality can be achieved by the researcher or the implementer. Also, possible changes in the original design of the structure might create other possibilities to target rehabilitation procedures on different parts of the body. Supplied game algorithms with pre-built user interface and interaction control scripts may also be easily adapted to these changes.

In general, as tried to be mentioned earlier, most of the robotic rehabilitation systems are expensive and inaccessible to many people who requires them for treatment opportunities. In some cases, patients might be in need for more intensive rehabilitation programs and performing these rehabilitation procedures at home might be given as an efficient solution to this. Also considering new challenges such as Covid-19 pandemic, where homecare becomes a necessity, importance of portable and inexpensive systems increase undeniably. However due to the space requirements of the most commercial rehabilitation systems and their high costs, it is difficult to adapt rehabilitation systems capable of passive and active treatments for regular home usage.

Opposite to the existing systems, throughout the compact structural design and manufacturing of PARS, relatively cheaper components and simple control applications were utilized within the limits of selected components. Also provided virtual game software offers various potential cognitive effects for the treatment procedures. Enabling physiotherapists and healthcare professionals to produce and use proposed system directly, new works can be published with clinical studies. In addition, it is possible to use mechanical system in different ways in line with the experiences of physiotherapists and with the use of system components in different ways, such as using more than one system in bilateral rehabilitation scenarios. Also adapting different games, faster access to game therapy researches can be achieved. In this way, newer studies on the effect of game rehabilitation on physical rehabilitation therapy can be carried out more quickly with lower budgets.

In summary;

• PARS is a simple, compact rehabilitation device that aims to exercise upper extremity with gamification by following passive, active assistance and active resistance rehabilitation procedures.
• PARS is a low cost rehabilitation device that can be accessed and used by the majority by following instructions in this work even without having too much experience in interaction control or advance technical experience.
• PARS is adaptable to changes in terms of hardware and software in order to be utilized in different type of exercises or games.
• PARS can be used in custom game development for post stroke rehabilitation.

## Design files summary

**Table:** 

**Design file name**	**File type**	**Open source license**	**Location of the file**
Motor Holder	CAD file (.ipt,.stp) & STL file	CC BY 4.0	https://doi.org/10.17632/wrx22bn64h.2
Calibration Adapter	CAD file (.ipt,.stp) & STL file	CC BY 4.0	https://doi.org/10.17632/wrx22bn64h.2
Motor End Sensor Holder	CAD file (.ipt,.stp) & STL file	CC BY 4.0	https://doi.org/10.17632/wrx22bn64h.2
Handle End Sensor Holder	CAD file (.ipt,.stp) & STL file	CC BY 4.0	https://doi.org/10.17632/wrx22bn64h.2
Handle	CAD file (.ipt,.stp) & STL file	CC BY 4.0	https://doi.org/10.17632/wrx22bn64h.2
Bearing Inner	CAD file (.ipt,.stp) & STL file	CC BY 4.0	https://doi.org/10.17632/wrx22bn64h.2
Load Cell Amplifier Circuit	fig & Fritzing model (.fzz)	CC BY 4.0	https://doi.org/10.17632/wrx22bn64h.2
Wireless Communication Circuit	fig & Fritzing model (.fzz)	CC BY 4.0	https://doi.org/10.17632/wrx22bn64h.2
Controller and Driver Circuit	fig & Fritzing model (.fzz)	CC BY 4.0	https://doi.org/10.17632/wrx22bn64h.2
Calib	.ino	CC BY 4.0	https://doi.org/10.17632/wrx22bn64h.2
Esp_udp	.ino	CC BY 4.0	https://doi.org/10.17632/wrx22bn64h.2
Velocity Control Tuning Model	slx	CC BY 4.0	https://doi.org/10.17632/wrx22bn64h.2
Velocity Control Deployed Model	slx,.hex	CC BY 4.0	https://doi.org/10.17632/wrx22bn64h.2
Upper Arm Rehab Game	Unity code	CC BY 4.0	https://doi.org/10.17632/wrx22bn64h.2
Upper Arm Rehab Game	Built executable file (RAR)	CC BY 4.0	https://doi.org/10.17632/wrx22bn64h.2
Config	.json	CC BY 4.0	https://doi.org/10.17632/wrx22bn64h.2
Pars Usage Video	.avi	CC BY 4.0	https://doi.org/10.17632/wrx22bn64h.2
Chip Test Video	.avi	CC BY 4.0	https://doi.org/10.17632/wrx22bn64h.2

### Mechanical design files

**Fig. 3:**
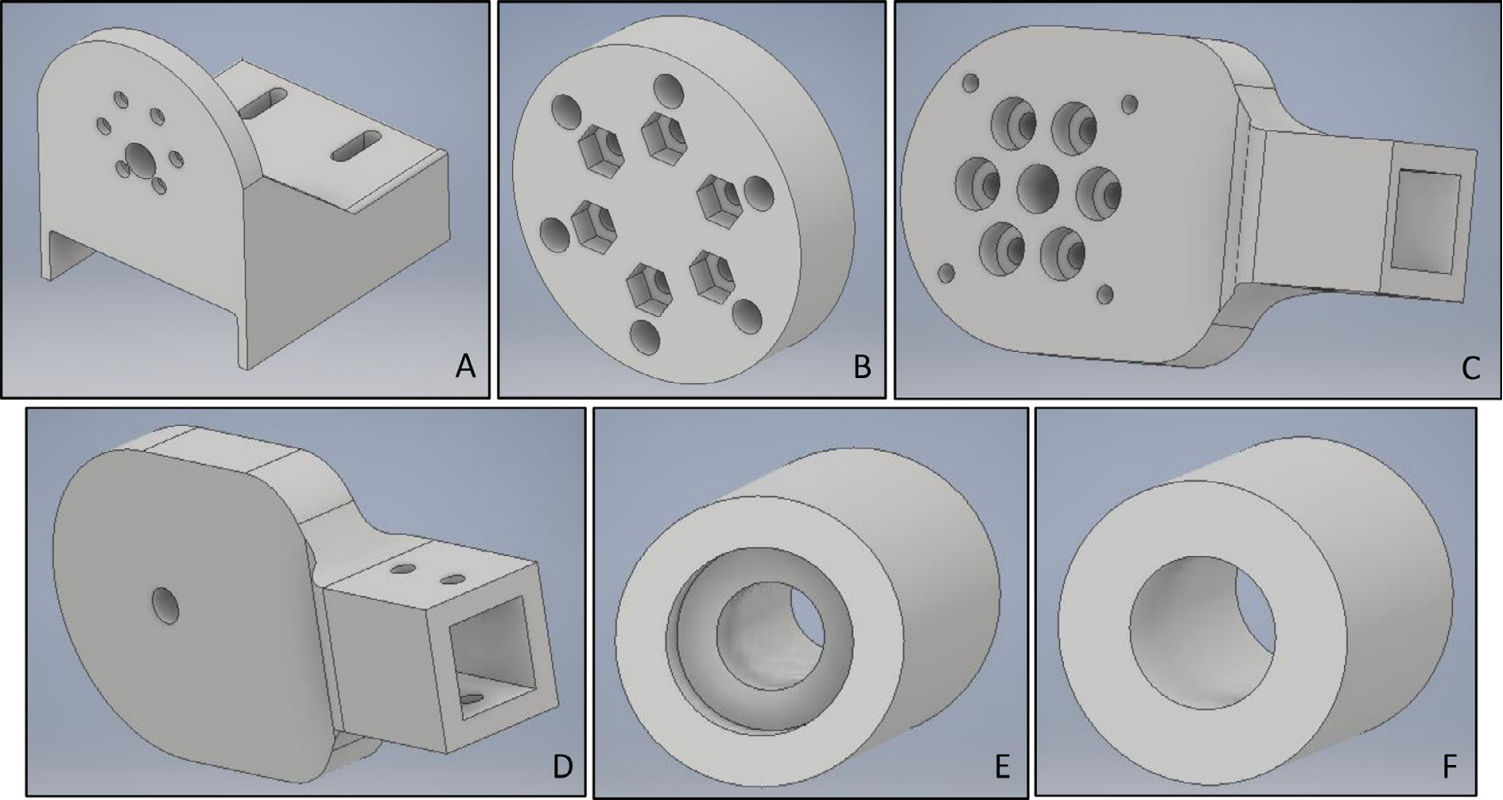
CAD designs (A) Motor holder (B) Calibration adapter (C) Motor end sensor holder (D) Handle end sensor holder (E) Handle (F) Bearing inner.

**Motor Holder (**Fig. 3**A):** This component is the main base for the systems actuator. It was designed to be assembled on a 40x80 aluminum extrusion profile. An alternative way to use this component is to cut off side fittings and directly connecting it to a stable ground without having the profile, yet there would be a challenge on calibration procedure, where the system should be rotated 90 degrees. In its current form, the system is adaptable to be used as the fixed calibration system. It can be used without an attached actuator together with “Calibration Adapter” during the calibration process, since the system must stand still during calibration. “Motor Holder” was manufactured via rapid prototyping using ABS material. It should be noted that printing was carried out with an infill density of 100%, due to the fact that it needs high strength for possible high loads on the component.

**Calibration Adapter (**Fig. 3**B):** This component is utilized during load cell calibration process. It was designed to be connected on the “Motor Holder”. During load cell calibration process, this component serves as the connection between “Motor Holder” and the “Force Link”, where the latter is the combination of “Motor End Sensor Holder”, “Handle End Sensor Holder” and the load cell itself. Thanks to this component, “Force Link” becomes stationery and required measurements can be carried out. It has six M3 nut shaped holes, which must be filled with the nuts after component manufacturing. “Calibration Adapter” was manufactured via rapid prototyping using ABS material with an infill density of 60%.

**Motor End Sensor Holder (**Fig. 3**C):** This component is one of the holders for the load cell. It is closer to the rotation axis. It carries required electronic components (“Load Cell Amplifier Circuit” and “Wireless Communication Circuit”). It is directly connected to OEM “Aluminum Mounting Hub” component, which is also connected to the actuator itself. The force intention comes from the user and the counter-torque from the actuator on the system is directly transferred on this component, thus strength of the material becomes important. This component was manufactured via rapid prototyping using ABS with an infill density of 100%.

**Handle End Sensor Holder (**Fig. 3**D):** This component is the other holder of the load cell, through which the user force intention is transferred to the load cell on the system. The sensing side of the load cell was fitted on this component and the handle was also connected to it. Due to the strength requirement of the component, infill density in manufacturing is recommended as 100%.

**Handle (**Fig. 3**E):** This component is the main interaction part between the user and PARS. It connects to the “Handle End Sensor Holder” and the user force is transferred through it. In order to have a comfortable usage during rehabilitation procedures, handle was designed to have a passive rotational degree of freedom. In this design, there are rooms for 10x26x8 bearings in both ends. “Handle” was manufactured via rapid prototyping using ABS with an infill density of 70%.

**Bearing Inner (**Fig. 3**F):** This component is placed in the bearing hole in order to keep the bearing stable. Also, M5 bolt is placed inside the component, eventually fixed connection between the handle and the “Handle End Sensor Holder” is acquired in this way. Since the component is relatively small and carries some portion of the load, infill density is recommended to be 100% during manufacturing.

### Electronic design files

**Fig. 4:**
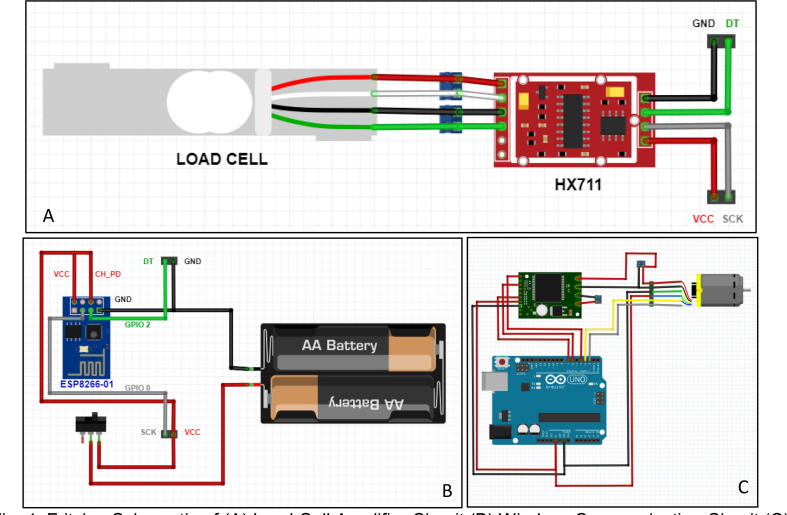
Fritzing schematic of (A) Load cell amplifier circuit (B) Wireless communication circuit (C) Controller and driver circuit.

**Load Cell Amplifier Circuit (**Fig. 4**A):** Small voltage difference that is produced in load cell measurements under the load needs to be amplified by using load cell amplifier board, HX711. Since every component should be organized, load cell amplifier circuit was assembled on a perforated board via soldering. Other components needed for this circuit assembly are two terminals and two headers.

**Wireless Communication Circuit (**Fig. 4**B):** ESP8266-01 is a small serial Wi-Fi wireless transceiver module with a built-in microcontroller and Wi-Fi capability, thus, it is convenient to use in proposed system. Assembled circuit is connected to the “Load Cell Amplifier Circuit” with two headers so that overall circuit would be duplex. Electrical power is transferred from the circuit on top (Wireless Communication Circuit) to the circuit beneath (“Load Cell Amplifier Circuit”) and output data of amplifier (HX711) is received from the circuit beneath to the circuit on top via specific headers. Simple on/off switch was also added to the circuit, which gives electronic board the capability to be turned on and off.

**Controller and Driver Circuit (**Fig. 4**C):** The main controller that sends commands to drive brushed DC motor via motor driver and reads built-in encoder pulses was selected to be an Arduino Uno microcontroller board. In order to have rigid and safe connections, all of the components were assembled together to form a shield like structure and soldered on a perforated board.

### Software design files

**Calib.ino (Appendix A1):** Arduino code for load cell calibration can be found as an uploaded document in given repository. This code is mainly based on the open source library, “HX711.h”, developed by github user “bogde” [36]. Calibration procedure mainly includes interpreting acquired data in proper measurement units for the task, such as grams or ounces. “Calib.ino” software was also initially developed by “Nathan Seidle” of SparkFun Electronics in 2014 [37] and modified by the authors and uploaded to the given repository. During the calibration process, the system should be executed with this code and “calibration_factor” variable should be determined. This value can be used in the system later.

**Esp_udp.ino (Appendix A2):** Acquired load cell data should be interpreted by using an open source “HX711.h” library, and then transferred to the software that is executed on Unity game environment. ESP8266-01 has a microcontroller on its embedded system together with the Wi-Fi capabilities. Load cell data, which is amplified by HX711 board, is read by the supplied embedded code, and the information is streamed with UDP protocol via Wi-Fi. In order to carry out the procedure, local network should be created and its SSID/password should be hardcoded to the embedded code. Also UDP port should be defined, which will be known in gaming environment usage. Basically this code provides simple load cell and UDP protocol reading.

It should also be noted that in order to use ESP8266-01 with Arduino integrated development environment (IDE), board should be installed to IDE.

**Velocity Control Tuning Model:** This model was built with Matlab/Simulink environment by using “Simulink Support Package for Arduino Hardware” package, which can be added to Matlab through add-ons in the software. By using this support package, it is possible to have proper real time communication between Arduino and Simulink model. It is very convenient to use it due to the advantage of building control model easily and tuning controller parameters in real time. Supplied model can be used with Matlab R2019a or its later version since the model includes “External Interrupt” block, which has been started to be supported in these versions of software.

Supplied tuning model has some visualization blocks, therefore the simulation should be XCP-Based, which can be configured by the setting “Communication Interface” to “XCP on Serial” under the “Hardware Implementation” tab.

**Velocity Control Deployed Model:** Similar to the “Velocity Control Tuning Model”, this model was also built with the same platform and support package. Control parameters that will be determined by utilizing previous model should be embedded to this model. It accepts reference velocity value via serial communication and streams number of rotations value of the actuators output shaft. This model should be deployed to Arduino by utilizing “Deployed to Hardware” option in “Hardware” tab in Simulink environment.

**Unity Model:** Unity model is the main program that executes gaming environment as well as outer loop of the control algorithm. It includes game flow, in which level difficulties, connection options and save criteria can be chosen. There exist “config.json” file, which collects all configuration information so that the user-defined default values can be changed before operating overall system. In all software pieces, detailed information was also supplied as the comments in software code. Also, their usages were explained in Section 6.

## Bill of materials summary

**Table:** 

**Designator**	**Component**	**Number**	**Cost per unit -currency**	**Total cost -** **currency**	**Source of materials**	**Material type**
	Arduino Uno	1	23.00 $	23.00 $	https://store-usa.arduino.cc/products/arduino-uno-rev3?selectedStore = us	Electronic
	ESP8266-01	1	1.97 $	1.97 $	https://www.ebay.com/itm/201501780189	Electronic
	Load Cell − 10 kg, Straight Bar (TAL220) + Load Cell Amplifier – HX711	1	8.99 $	8.99 $	https://www.amazon.com/dp/B07KZBDXJL/ref = twister_B099PQHX3L?_encoding = UTF8&th = 1	
	BATTERY LITHIUM 3 V6 1200mAh	1	2.30 $	2.30 $	https://www.ozdisan.com/bataryalar/sarj-edilemeyen-piller/lityum-piller/ER14250-2PF-3–6 V	
	131:1 Metal Gearmotor 37Dx73L mm 12 V with 64 CPR Encoder (Helical Pinion)	1	39.95 $	39.95 $	https://www.pololu.com/product/4756	
	VNH5019 Motor Driver Carrier	1	49.95 $	49.95 $	https://www.pololu.com/product/1451	Electronic
	Pololu Universal Aluminum Mounting Hub for 6 mm Shaft, M3 Holes (2-Pack)	1	7.95 $	7.95 $	https://www.pololu.com/product/1999	
	Perforated Board 4–5/16″ x 3–1/8″	1	6.33 $	6.33 $	https://www.amazon.com/Parts-Express-Perforated-Board-320–430/dp/B0002ZPUDK	
	2.54 mm Single Row Male Pin PCB Header Connector	1	0.14 $	0.14 $	https://www.amazon.com/Hotop-Pack-Single-Header-Connector/dp/B06XR8CV8P	
	2.54 mm Single Row Female PCB Header Connector	1	0.17 $	0.17 $	https://www.amazon.com/Qunqi-2–54 mm-Straight-Connector-Arduino/dp/B07CGGSDWF	
	Slide Switch − 90 Degree	1	1.85 $	1.85 $	https://www.amazon.com/Smal-SPDT-Slide-Switch-Degree/dp/B0752WJL6V	
	2 Pin Terminal Block Connectors	3	0.31 $	0.93 $	https://www.amazon.com/50Pcs-Terminal-Connectors-2–54 mm-Connector/dp/B07C9ZCSBY	
	40×80 Aluminum Extrusion Profile	0.3 m	39.71 $	13.58 $	https://www.aluminium-profile.co.uk/40x80-itm-aluminium-profile-ir02604	
	4040 Series M6 T-Nut Sliding Nut T-Slot Nut for Aluminum Extrusion Profile	4	1.25 $	5.00 $	https://www.amazon.com/PZRT-Sliding-Aluminum-Extrusion-Profile/dp/B07PGN1LDP?th = 1	
	6000-2RS 6000-ZZ Radial Ball Bearing 10×26×8	2	1.07 $	2.14 $	https://thebigbearingstore.com/6000-2rs-6000-zz-radial-ball-bearing-10x26x8/	
	M6×25mm Stainless Steel Hex Socket Cap Screws Head Key Bolts	4	0.50 $	2.00 $	https://www.amazon.com/M6x25mm-Stainless-Steel-Socket-Screws/dp/B011BNP1EE	
	M5x30mm Stainless Steel Hex Socket Head Cap Screw A4	1	0.29 $	0.29 $	https://www.thefastenerfactory.com.au/stainless-steel-hex-socket-head-cap-screw-a4-316-m5-x-30 mm-100pc	
	M5×0.8 mm Metric Coarse Thread Hexagon Nut, Stainless Steel 304	2	0.09 $	0.18 $	https://www.amazon.com/uxcell-Metric-Stainless-Hexagon-Silver/dp/B07H3X397L/	
	M4×20mm Stainless Steel Hex Socket Head Cap Screw A2	4	0.08 $	0.32 $	https://www.thefastenerfactory.com.au/stainless-steel-hex-socket-head-cap-screw-m4-x-20 mm-100pc	
	M3×30mm Stainless Steel Hex Socket Head Cap	6	0.13 $	0.78 $	https://www.thefastenerfactory.com.au/stainless-steel-hex-socket-head-cap-screw-m3-x-30 mm-100pc	
	M3×16mm Stainless Steel Hex Socket Head Cap	6	0.05 $	0.30 $	https://www.thefastenerfactory.com.au/stainless-steel-hex-socket-head-cap-screw-m3-x-16 mm-100pc	
	M3×12mm Stainless Steel Hex Socket Head Cap	6	0.05 $	0.30 $	https://www.thefastenerfactory.com.au/stainless-steel-hex-socket-head-cap-screw-m3-x-12 mm-100pc	
	M3×9mm Thread Button Head Hex Socket Cap Screw Bolt	6	0.09 $	0.54 $	https://www.amazon.com/uxcell-M3x9mm-Thread-Button-Socket/dp/B01AXURX42	
	M3 Stainless Steel Hex Nut	12	0.02 $	0.24 $	https://www.thefastenerfactory.com.au/stainless-steel-hex-nut-m3-200pc	
	M2×15mm Alloy Steel Button Head Hex Socket Screws	4	0.16 $	0.64 $	https://www.amazon.com/XunLiu-Grade-Button-Socket-Screws/dp/B0756VQBKQ?th = 1	
	M2-0.4 Thread Size, 4 mm Width Across Flats, 1.6 mm Thick	4	0.07 $	0.28 $	https://www.amazon.com/Finish-Metric-M2-0–4-Thread-Across/dp/B009EFMQKY	

## Build instructions

PARS is an electro-mechanical system setup combined with a gaming software. It is crucial for the load cell to be calibrated prior to the operation. Therefore, two different setups should be assembled sequentially. All of the required components for both assemblies were supplied in the repository.

**Fig. 5:**
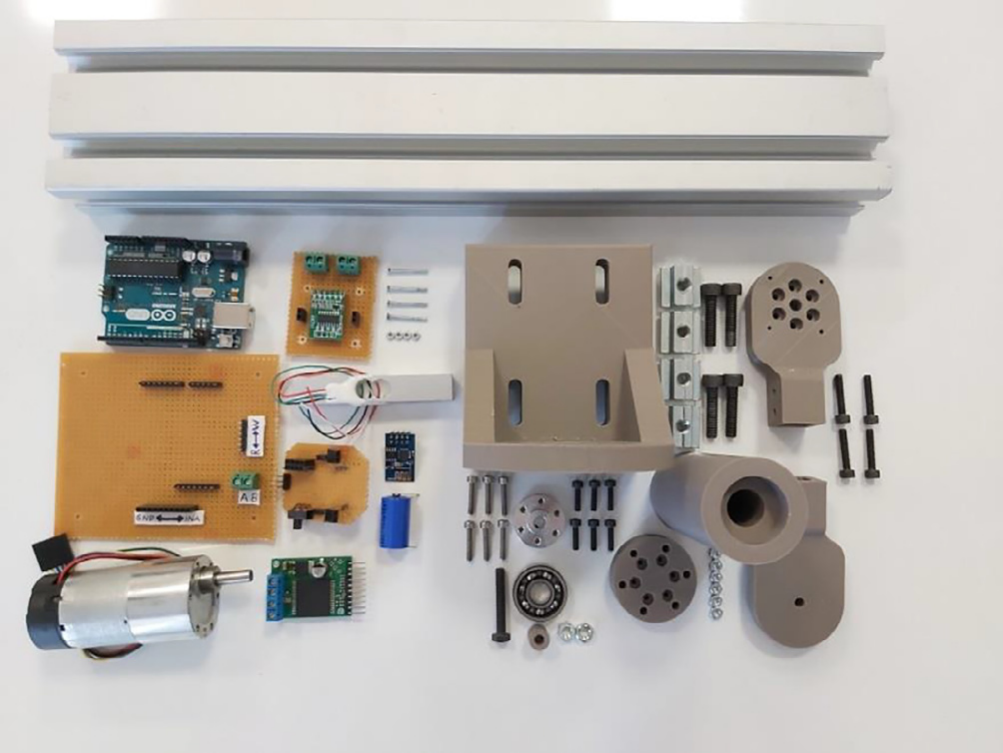
All designed, manufactured and ready parts.

PARS can be easily manufactured via rapid prototyping and using basic components that were mentioned in bill of materials table. Throughout this study, components were printed out with Zortrax M200 rapid prototyping machine by using ABS material varying infill densities with respect to the component loading conditions. All the components that were used in the hardware can be seen in Fig. 5.

### Electronic components

**Fig. 6:**
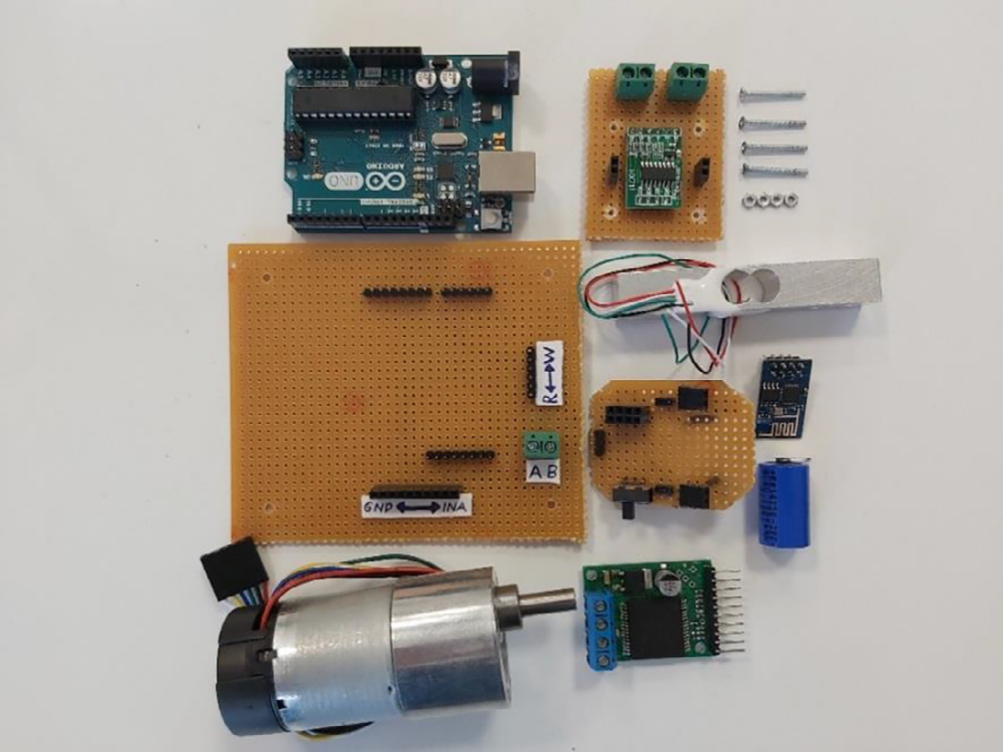
All electronic parts.

Mainly there exist three different electronic assembly modules in the system, labeled as “Controller and Driver Circuit”, “Load Cell Amplifier Circuit” and “Wireless Communication Circuit” (Fig. 6). Arduino Uno is the main controller in the system for controlling brushed DC motor. In order to drive this actuator, VNH5019 motor driver is used in the system.

In this scenario Arduino Uno generates needed signals for direction and velocity. In order to adjust the voltage on the actuator, rotational velocity, PWM signal (PWM pin) is generated. Similarly two different digital signals (InA and InB pins) are generated by the controller for the actuator rotation direction. With respect to the definition in VNH5019 motor driver datasheet [38], opposite digital signals to these rotation pins define the direction. The logic behind direction criteria was created in Matlab/Simulink model.

**Fig. 7:**
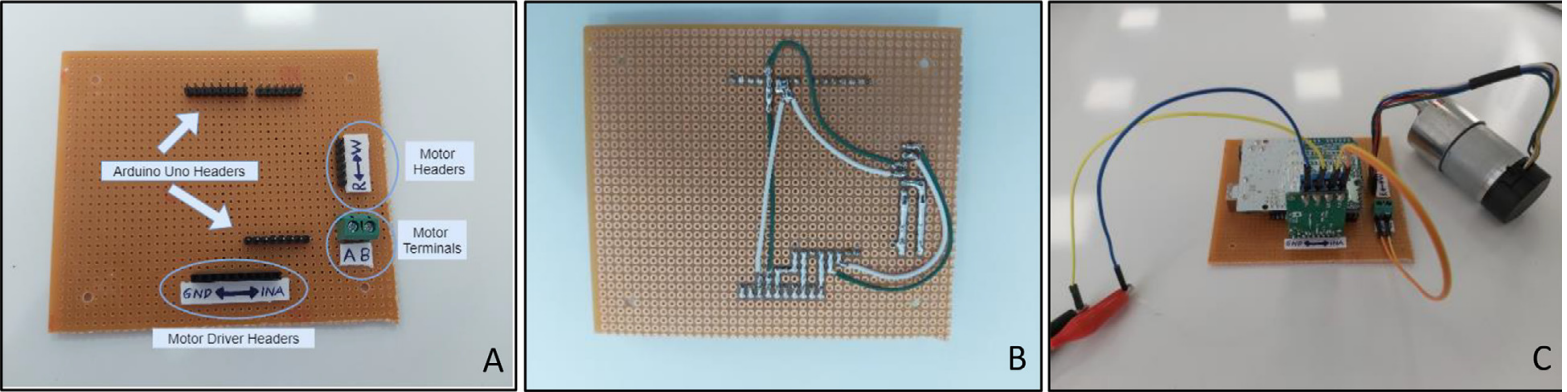
Controller and driver circuit (A) Top view (B) Bottom view (C) Together with components.

“Controller and Driver Circuit” was designed and soldered on a perforated board. Arduino Uno, motor driver, and actuator connections were combined in a single perforated board (Fig. 7A). Connections were soldered under the perforated board directly by using pin connections (Fig. 7B). At the end the components were assembled together on this simple electronic card (Fig. 7C).

**Fig. 8:**
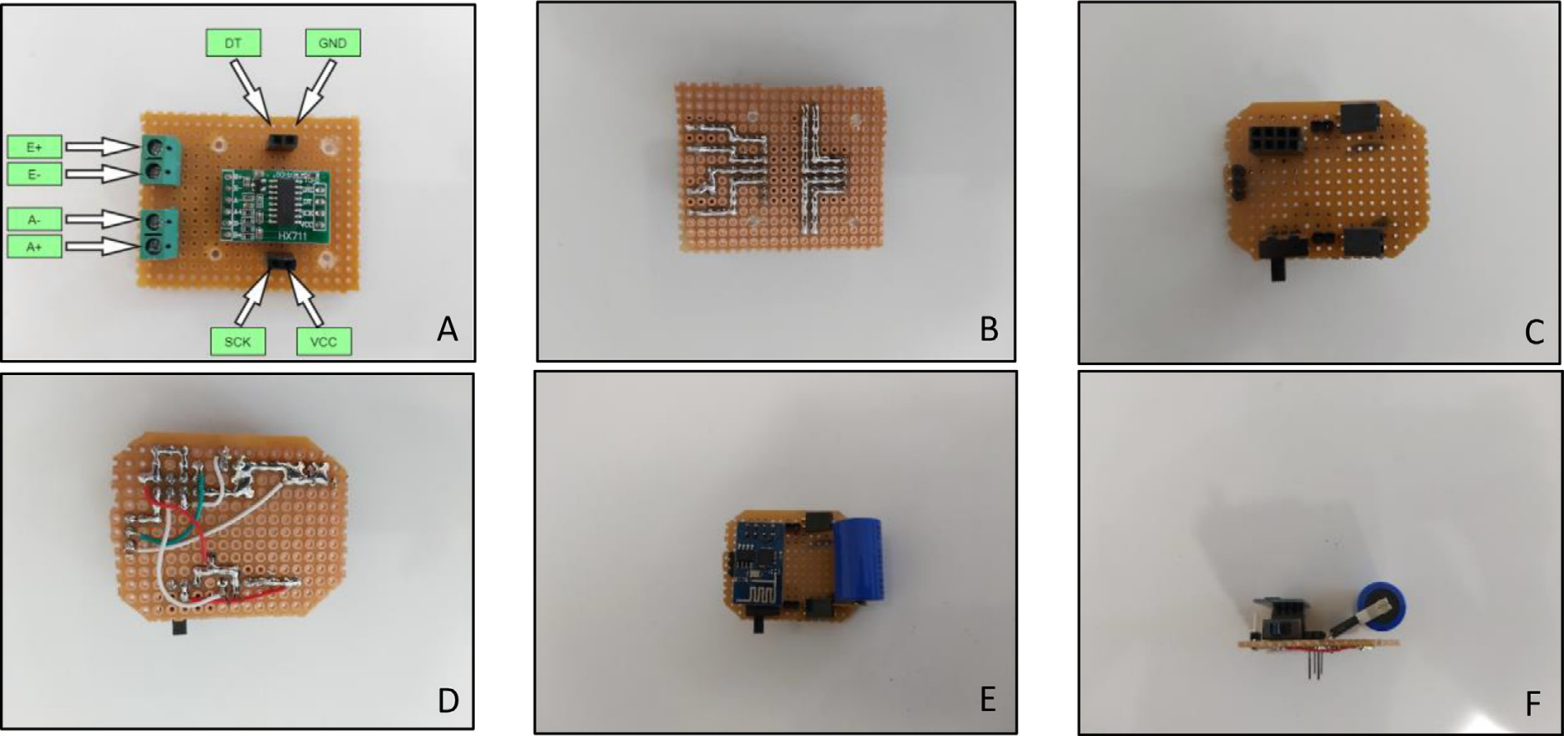
(A) Load cell amplifier circuit top view (B) Load cell amplifier circuit bottom view (C) Wireless communication circuit top view without components (D) Wireless communication circuit bottom view (E) Wireless communication circuit top view with components (F) Wireless communication circuit side view with components.

“Load Cell Amplifier Circuit” and “Wireless Communication Circuit” were placed together on the handle, in order to read load cell values and transmit interpreted data to the game environment wirelessly. Since load cell creates very tiny voltage difference in its own, an amplifier circuit was used. HX711 is a widely used component, thus it was selected to be used in this system. In terms of wireless communication, ESP8266-01 was used in order to have a built-in interpreter and data transmitter. Connections in this electronic can be seen in Fig. 8. This part was produced as duplex in order to have the component as compact as possible. 1200 mAh 3.6 V lithium battery was utilized as a power supply for the components.

### Mechanical components

As mentioned before, it is crucial for the load cell to be calibrated prior to the system operation in order to have proper measurements. Thus, proposed mechanical system has a fixed setup mode opportunity to take measurements from the load cell by applying known forces. During this procedure, embedded Arduino code for calibration (calib.ino) should be downloaded to the hardware. It should be noted that main rehabilitation setup that includes system actuator differs from the calibration setup. In this mode, instead of assembling actuator to the system, force link should be fixed to the motor housing.

**Fig. 9:**
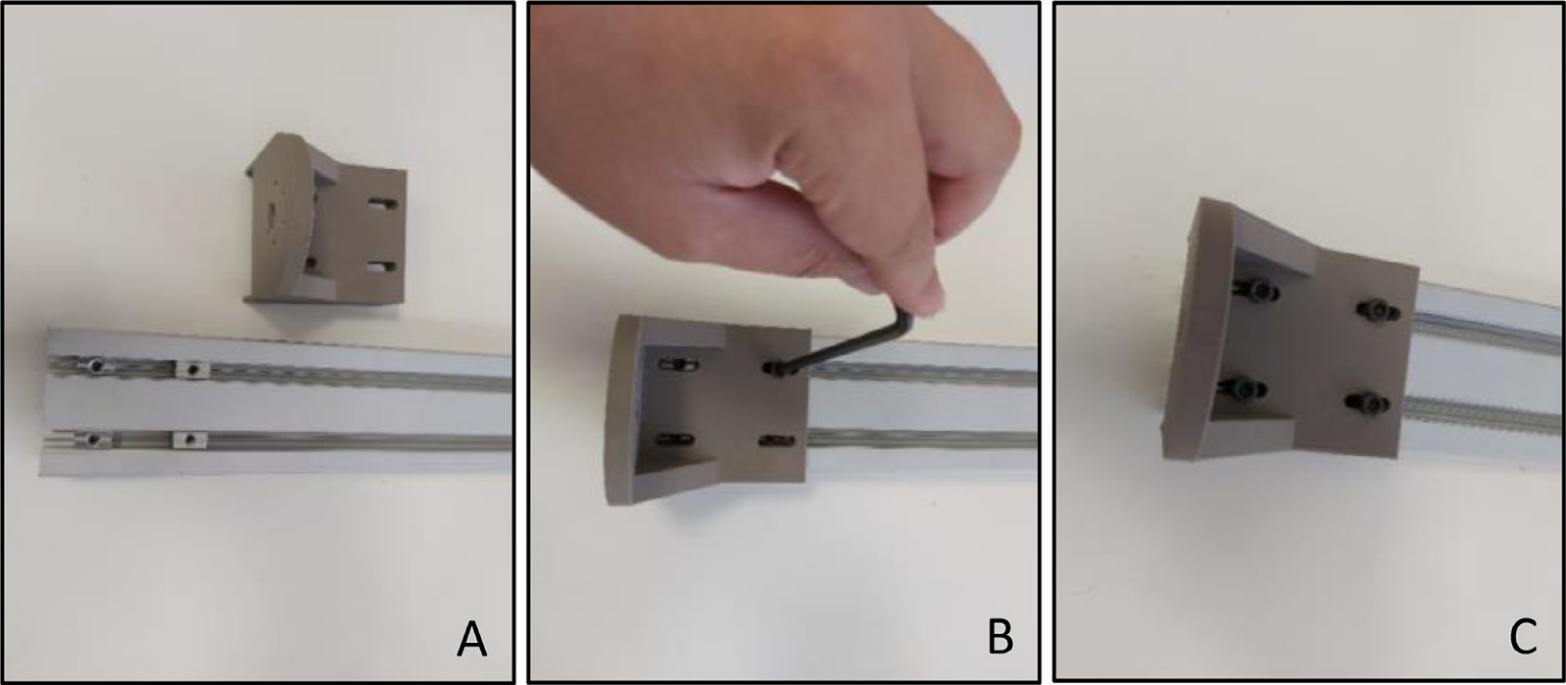
Motor housing with sigma profile (A) Before fixing (B) During fixing (C) After fixing.

In order to start calibration mode assembly of the system, four M6 t-slot nuts were placed inside the slots on the base (Fig. 9A) as a first step. Motor housing was mounted in such a way that placed nuts and assembly holes are coincident (Fig. 9B). M6×25 bolts were tightened with an Allen key of metric hex size 5 mm (3/16′’) (Fig. 9C).

**Fig. 10:**
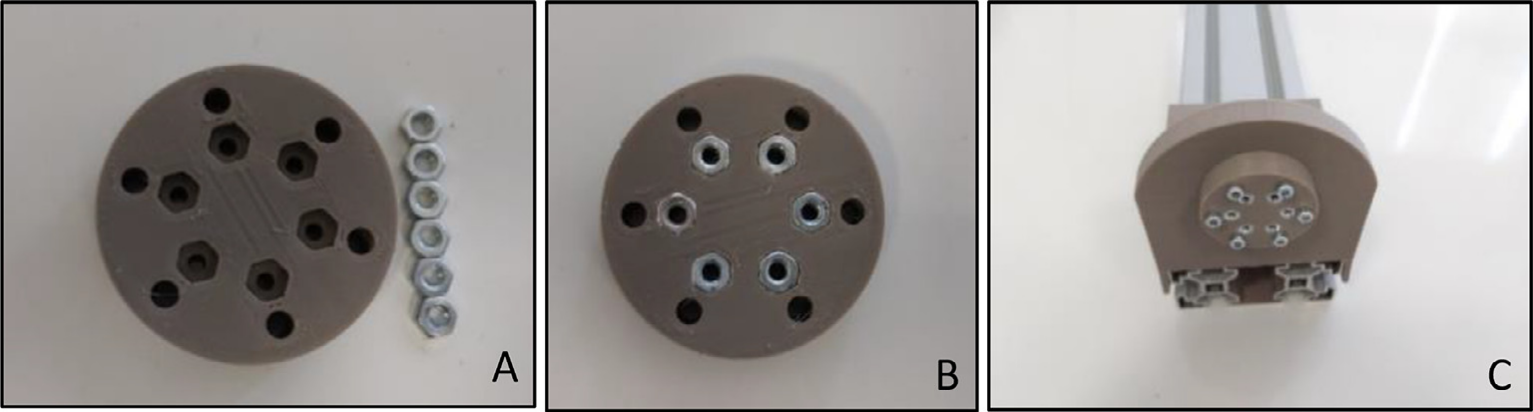
Calibration adapter (A) Manufactured (B) With M3 nuts (C) Assembled in motor holder.

“Calibration Adapter” was designed and manufactured to have a fixed base for the “Force Link” carrying known weights (Fig. 10A) during the calibration procedure. As seen in Fig. 10 there exist six holes with M3 nut geometry on the component. In order to fix the “Force Link” six M3 nuts should be placed in these holes (Fig. 10B). Outer holes are used as a bolt and nut connection via M3x30 bolts and M3 nuts in order to create a rigid connection between “motor housing” and “Calibration Adapter” (Fig. 10C).

**Fig. 11:**
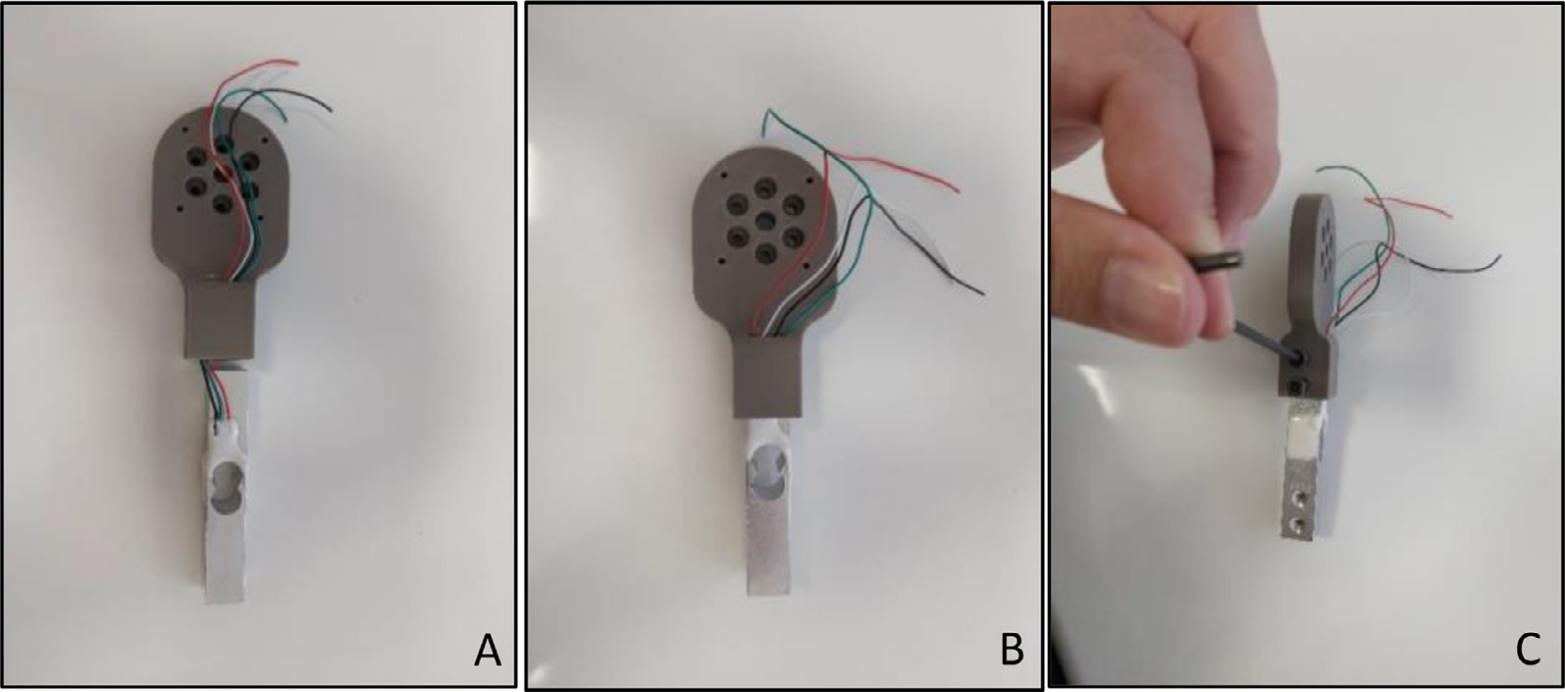
Motor end sensor holder (A) Cable reeving (B) placing load cell in rectangle hole (C) Fixing with allen.

“Force Link” that carries the load cell itself can be assembled separately. Cables of the load cell should be reeved through the small hole on the “Motor End Sensor Holder” (Fig. 11A) and the load cell should be shrink fit until the end of the rectangle shaped hole on “Motor End Sensor Holder” (Fig. 11B). As the last step of the motor end of the “Force Link” assembly, M4x20 bolts should be tightened through corresponding holes with an Allen key of metric hex size 3 mm (7/64’’) (Fig. 11C).

**Fig. 12:**
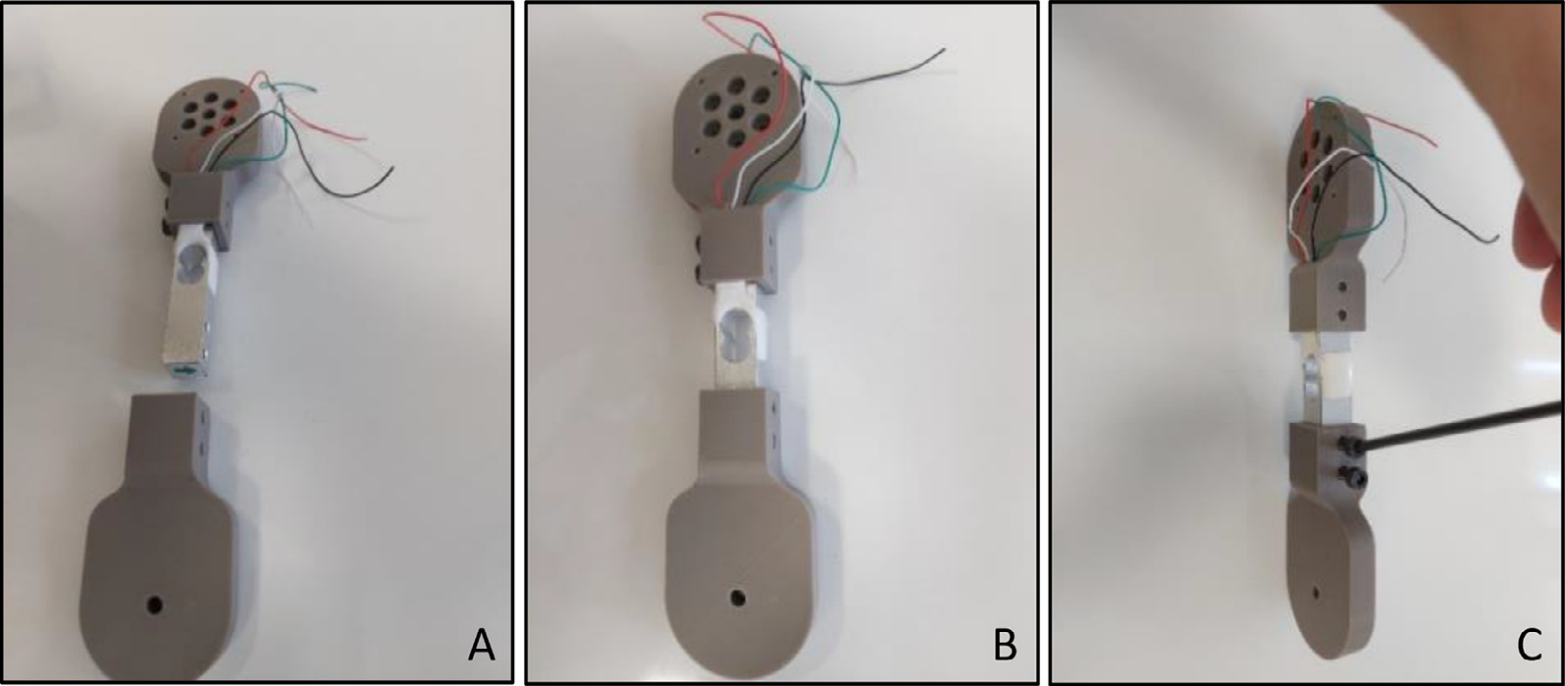
Handle end sensor holder (A) Direction (B) Placing load cell in rectangle hole (C) Fixing with allen.

Similar process should be applied to the “Handle End Sensor Holder” of the “Force Link”. Load cell should be pushed through the rectangle-shaped hole of the “Handle End Sensor Holder” (Fig. 12A and B), and M4×20 bolts should be tightened through corresponding holes with an Allen key of metric hex size 3 mm (7/64’’) (Fig. 12C).

**Fig. 13:**
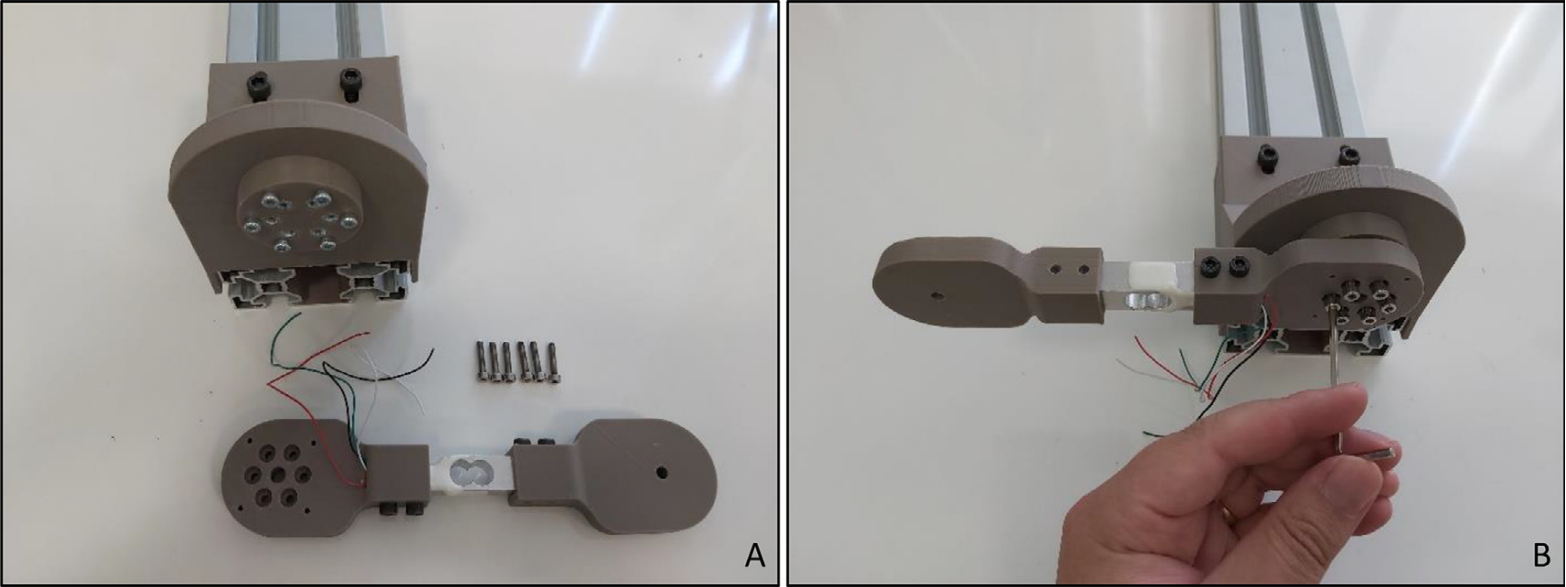
(A) Force link and calibration pieces (B) Fixing with allen.

Assembled “Force Link” should be fixed to the “Motor Holder” parallel to the ground by the help of “Calibration Adapter”. Six M3x16 bolts are needed for this operation (Fig. 13A). Using an Allen key of metric hex size 2.5 mm (3/32’’) the bolts should be tightened and the fixed connection should be completed (Fig. 13B).

**Fig. 14:**
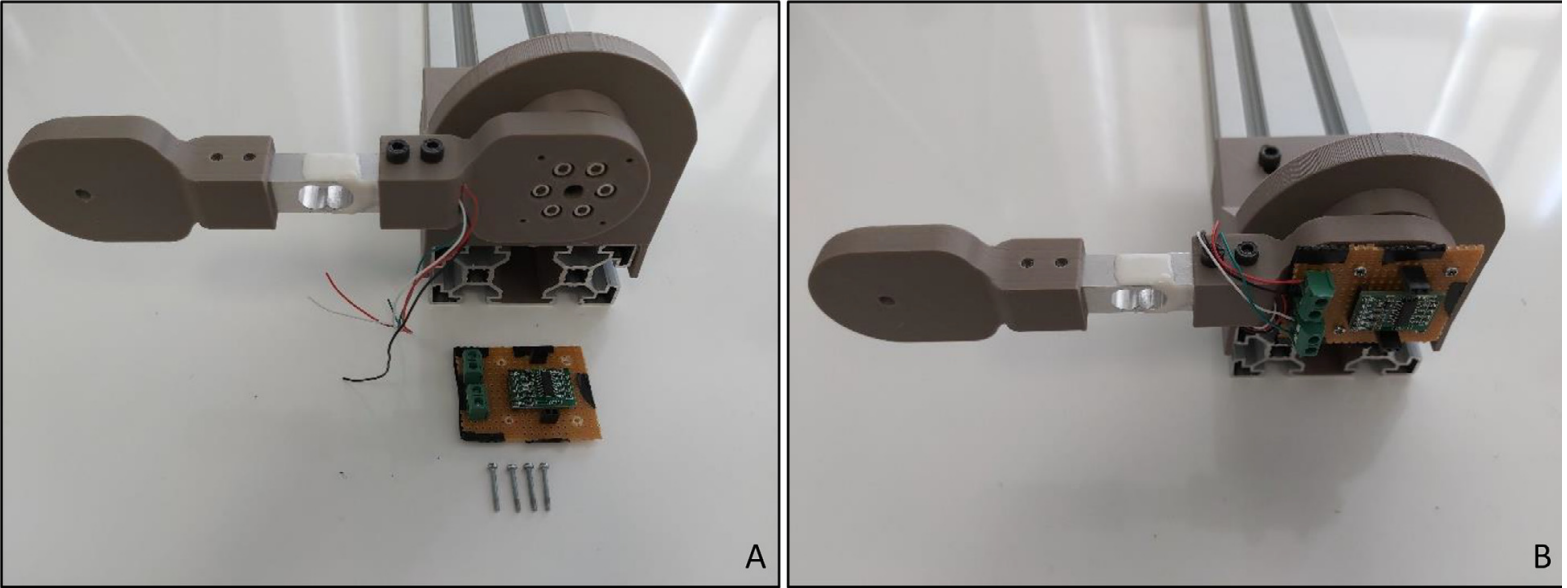
Fixed force link (A) without circuit (B) with circuit.

“Load Cell Amplifier Circuit” should be placed on the “Force Link” that is fixed to the “Motor Holder” oriented parallel to the ground. Due to the stationary pose in calibration procedure, four M2x15 bolts are sufficient to hold the board (Fig. 14A and B). After placing the circuit, load cell cables should be fixed on the terminals with the shown color code sequence (Fig. 14B).

As the “Force Link” was fixed parallel to the ground in this mode putting known weights on it would cause strain gauge on load cell to deform so that the measurements can be extracted. This configuration is an important check point, where the load cell calibration must be handled. The detailed information about the procedure was given in Section 6.

**Fig. 15:**
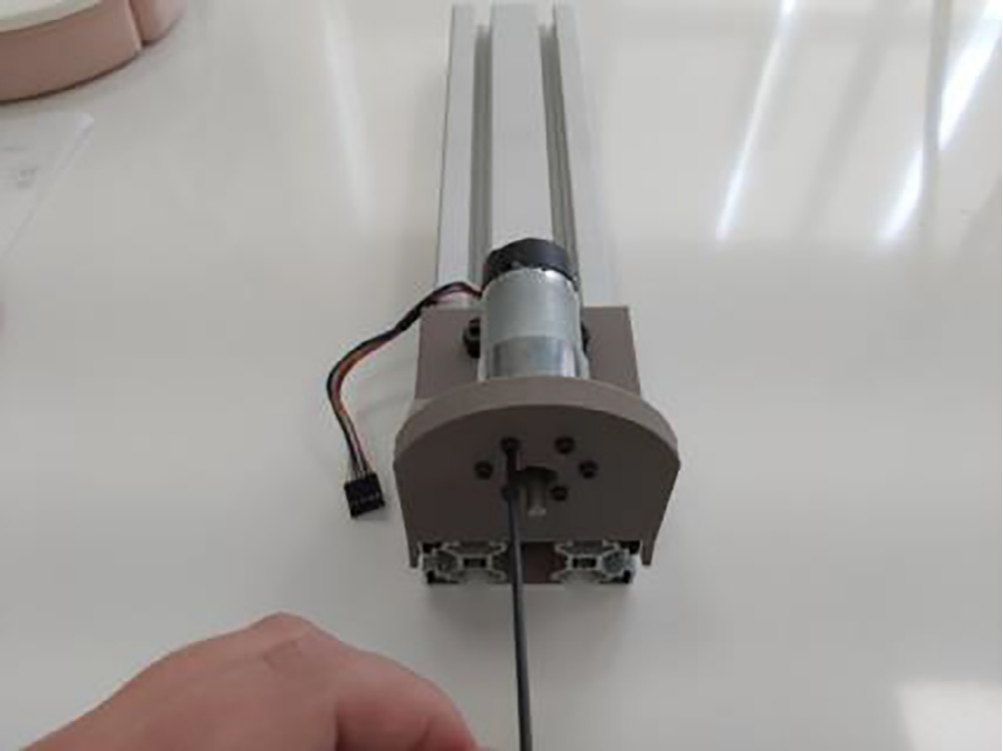
Motor holder with electric motor.

After the calibration process, “Calibration Adapter” should be removed from the system so that actuator assembly can be performed. For this procedure six M3x9 bolts are needed, which should be directly connected to the electric motor and they can be mounted with an Allen key of metric hex size 2.5 mm (3/32’’) (Fig. 15). Note that lengths of the bolts are pretty important here, due to the fact that if they are excessively long, actuator can be damaged.

**Fig. 16:**
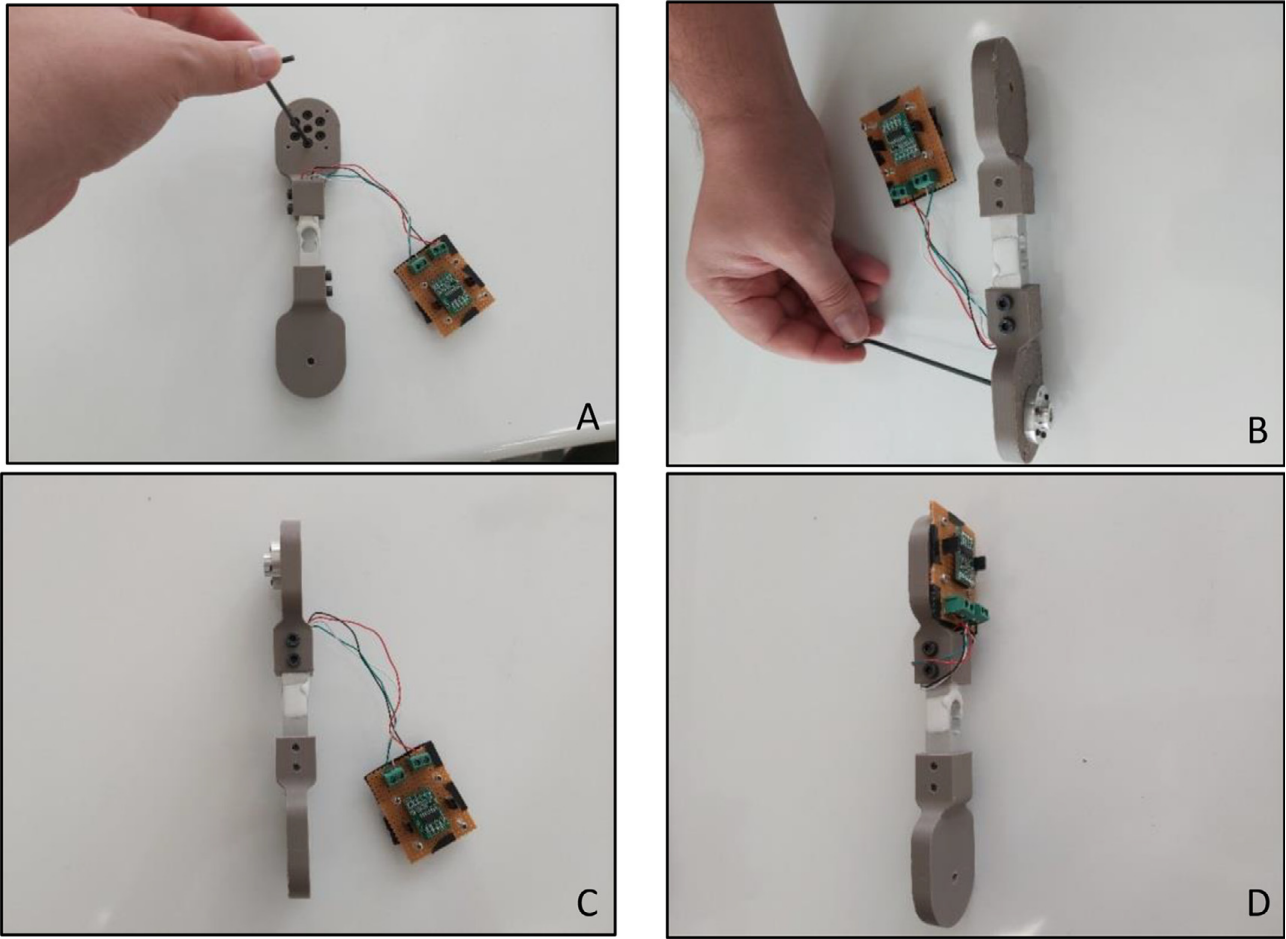
(A–D) Assembling motor mounting hub to force link.

In order to attach the “Force Link” to the actuator, “Motor Mounting Hub” should be connected to it. In order to connect them together with an Allen key of metric hex size 2.5 mm (3/32’’), four M3x12 bolts are needed (Fig. 16A, B, C). As in the calibration procedure, “Load Cell Amplifier Circuit” should be connected to the motor end of the “Force Link” (Fig. 16D). Therefore, four M2x15 bolts with M2 nuts are required due to the continuous rotation of the “Force Link” during the main operation.

**Fig. 17:**
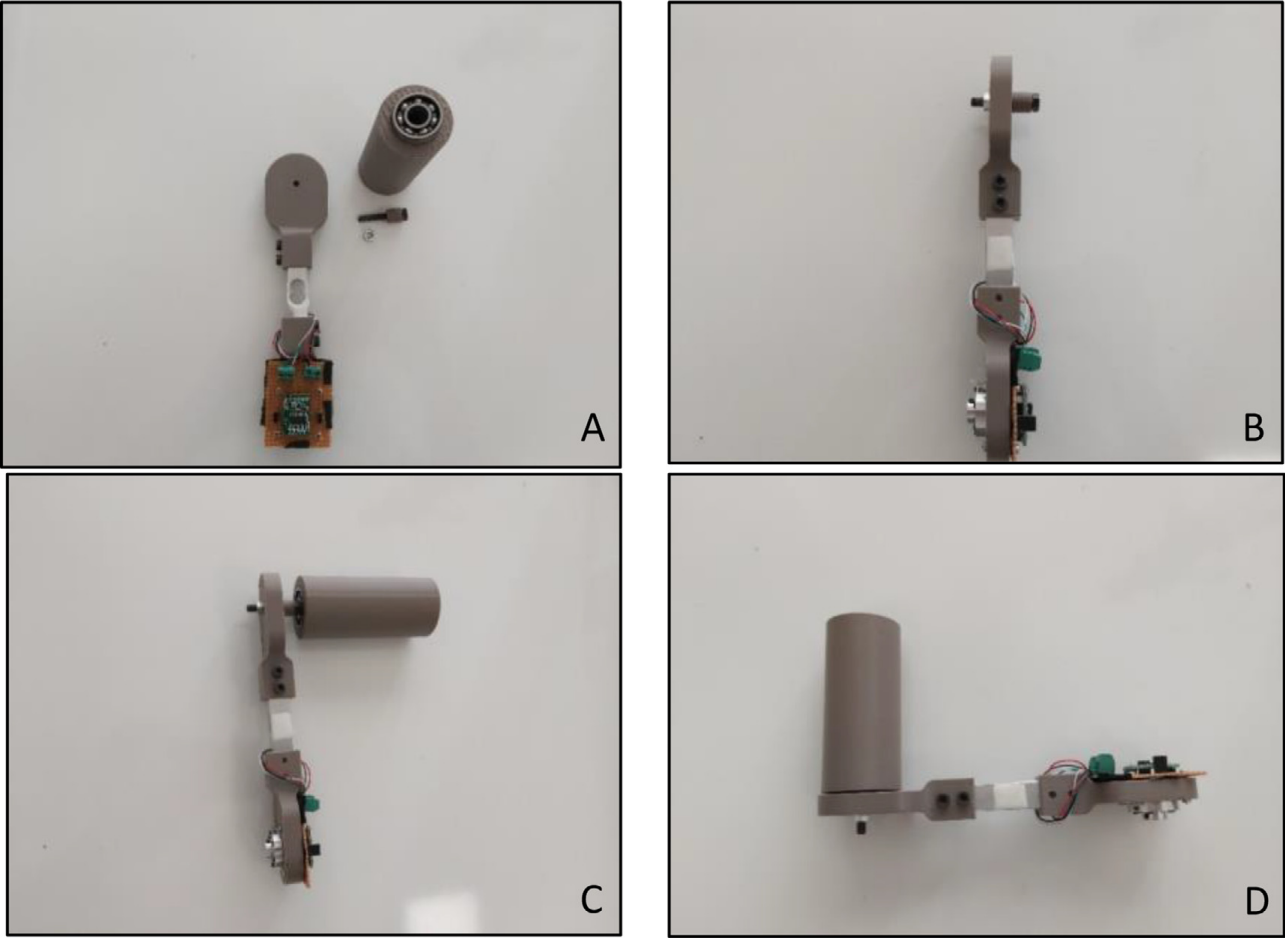
(A) Force link with required components (B) Bearing inner with M5×30 bolt (C) Putting handle on bearing inner (D) Overall handle with force link.

In order to have a comfortable usage during the operation, a handle with a passive rotation should be attached to the “Handle End Sensor Holder”. Firstly, 10x26x8 bearing should be shrink fitted to the “Handle”. Then “Bearing Inner” part should be passed into M5×30 bolt and fixed to the “Handle End Sensor Holder” with a bolt-nut connection (Fig. 17B). Handle should be fixed to the M5x30 bolt connection from the bearing side (Fig. 17C) so that passive rotation can be achieved (Fig. 17D). Alternatively, in order to have more rigid connection, it is also possible to use a longer bolt (M5×100) with two “Bearing Inner” parts and two 10x26x8 bearings. In this configuration, two bearings should be fixed to the both ends of the handle. Longer bolt will cause more rigid connection and resist to bending thanks to the additional bearing.

**Fig. 18:**
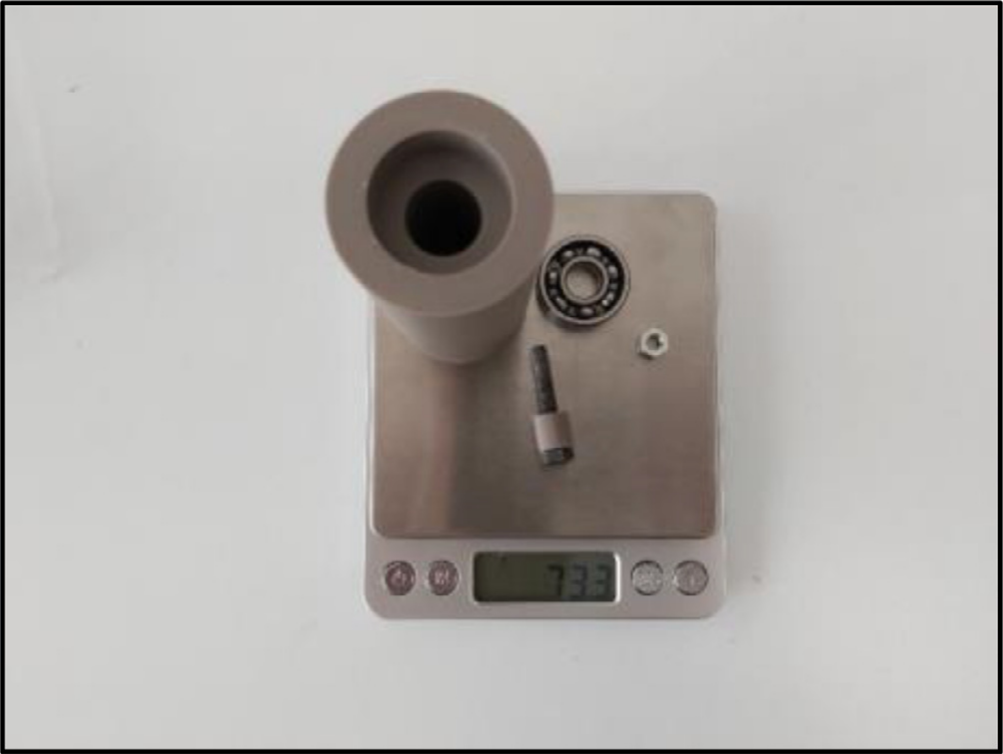
Total weight of the sensed side components.

During the operation of the system, weight on the sensed side of the load cell must be used in the gravity compensation model, thus the total weight of the sensed side components should be measured (Fig. 18).

**Fig. 19:**
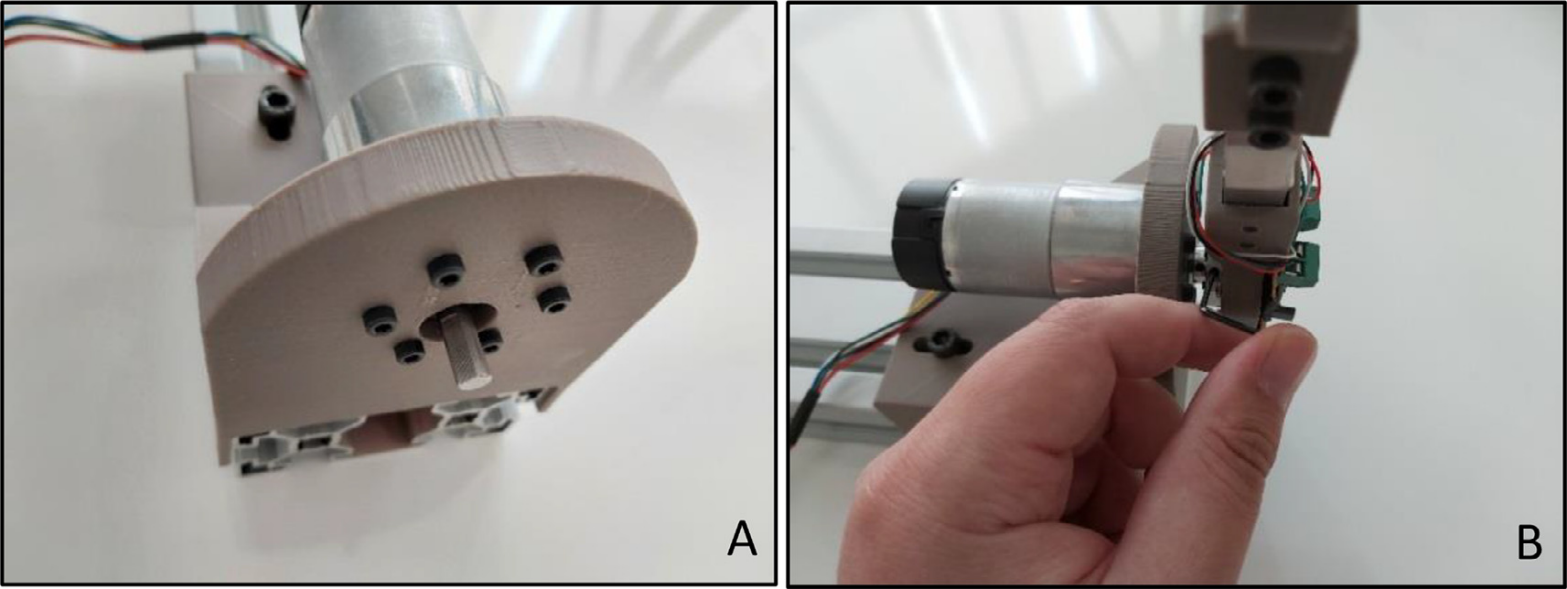
(A) “D” output shaft of motor (B) Setscrew connection on aluminum motor mounting hub.

Assembled “Force Link” with handle should be fixed to the “D” shaft of the actuator (Fig. 19A) by using setscrew connection on the aluminum motor mounting hub (Fig. 19B).

The electro-mechanical hardware can be easily built with the instructions described above. As a next step, overall system should be operated collaboratively with the supplied software so that rehabilitation procedures can be executed.

## Operation instructions

Before the operation of the complete system, important steps must be handled in prior. First of all, the load cell must be calibrated to convert strain gauge voltage difference to interpretable measurement units, in grams or ounces. This calibration process is pretty straightforward, where the supplied software with calibration mechanics is easily applicable. Another step to consider is to tune inner velocity loop for the electric motor by using supplied velocity control tuning model. In admittance control, inner motion loop is important and has a huge effect on the performance of overall interaction control. Since electric motors will be affected differently in terms of their structures control tuning model is also supplied. Thus it can be utilized in any conditions without having too much experience in actuator control.

### Load cell calibration

**Fig. 20:**
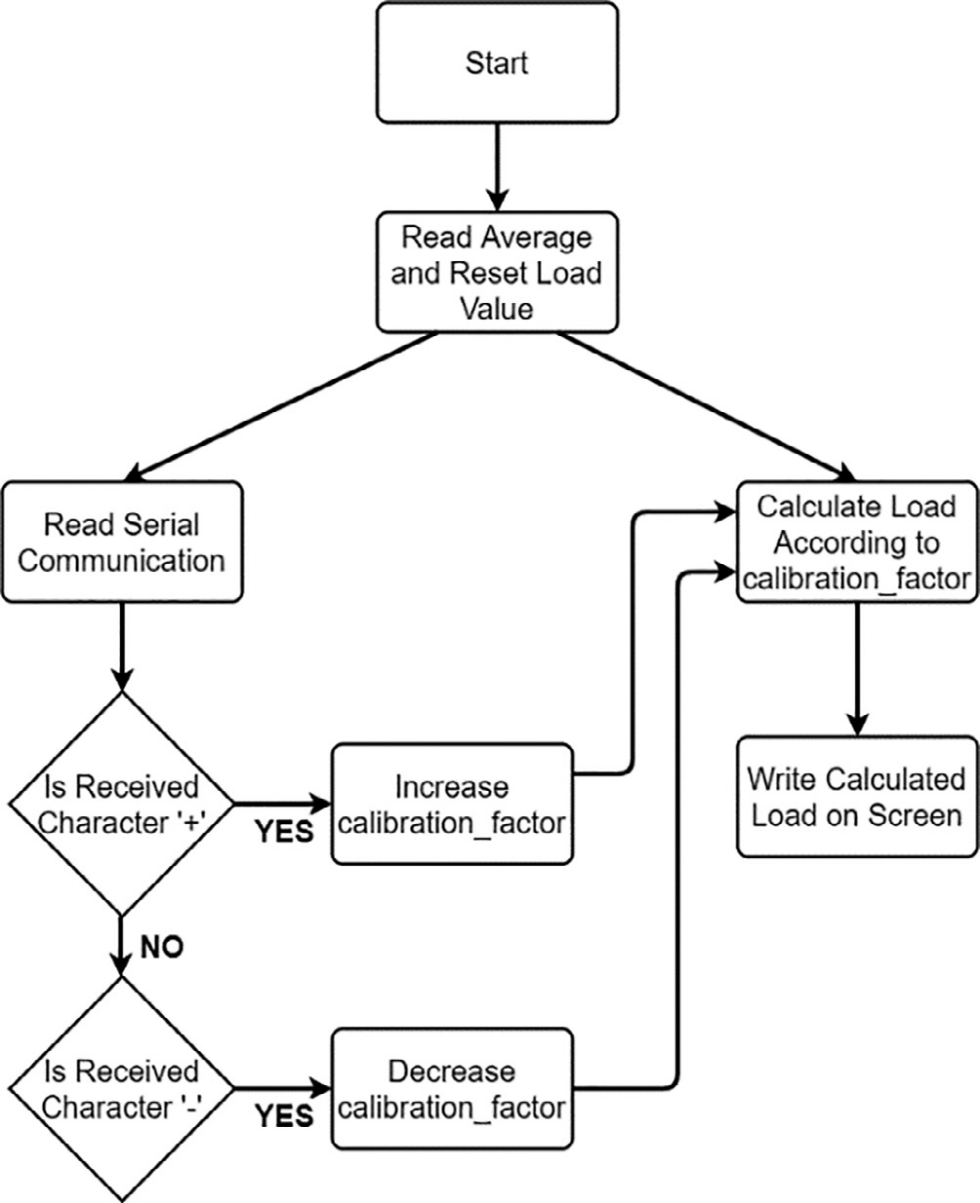
Flowchart of calibration code “calib.ino”.

In order to utilize calibration procedure once for the load cell, hardware should be brought to calibration ready assembly configuration as in Fig. 14. In such case, there will be no rotational movement in the system, thus “Wireless Communication Circuit” on the system is not needed to be assembled. In order to calibrate equipped load cell, Arduino Uno can directly be connected to the load cell amplifier with the connections given in calib.ino embedded code that should be downloaded to Arduino Uno. Flowchart of the embedded calibration code can be seen in Fig. 20.

**Fig. 21:**
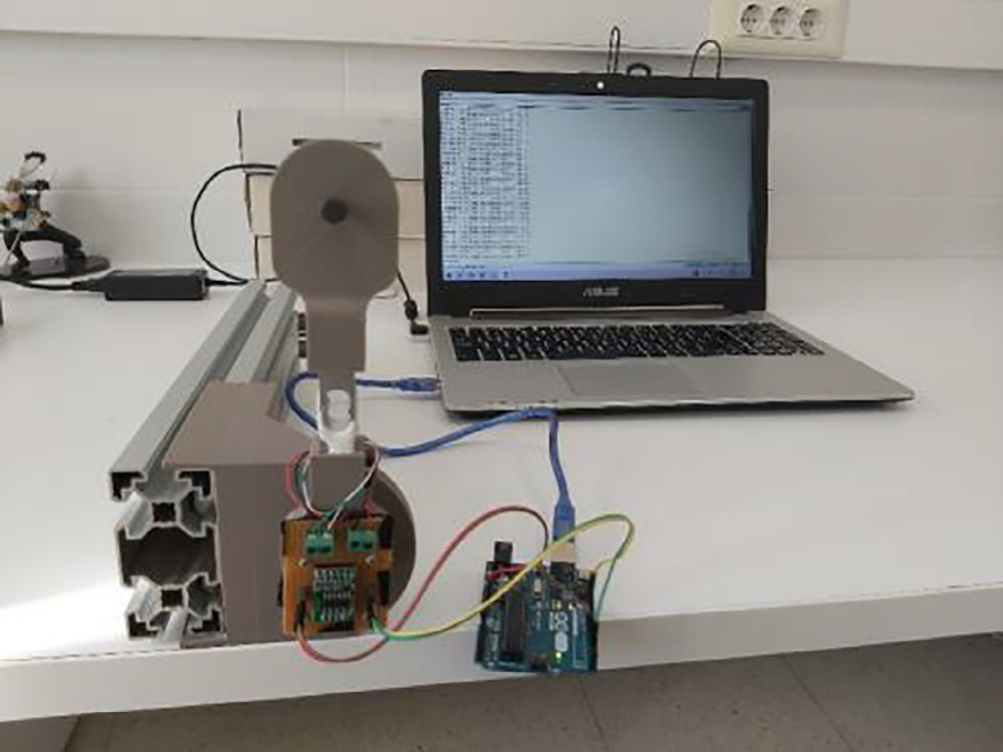
Initial pose of load cell calibration procedure.

At this point, it is important to note that, when Arduino Uno is powered on with the USB of a PC, there must be no load on the load cell for a few seconds for self-resetting. Thus base of the system should be rotated 90 degrees as in Fig. 21.

**Fig. 22:**
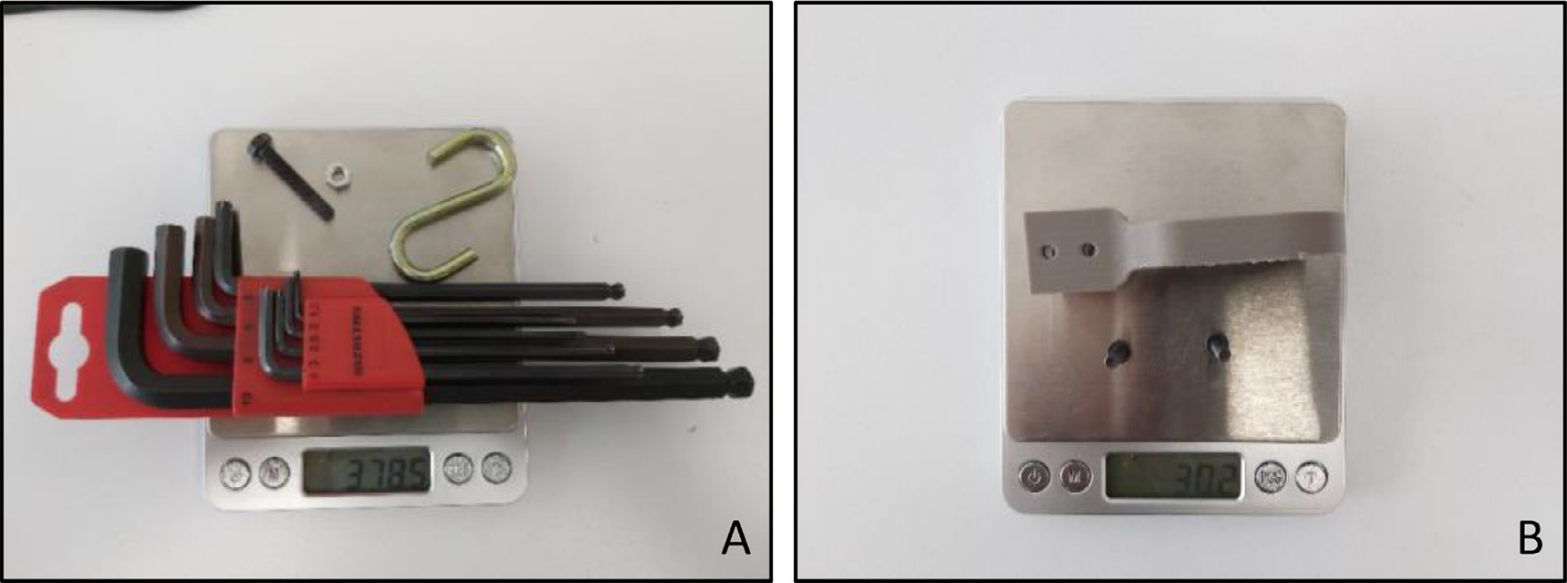
(A) Extra weights that hanged on force link (B) Natural weights on sensed side.

Calibration factor of the load cell can be determined by the help of known weights that are hanged on the handle side of the “Force Link”. Apart from the extra weights that were hanged on the system (Fig. 22A), weights of the actual assembly parts on the sensed side must also be known (Fig. 22B).

**Fig. 23:**
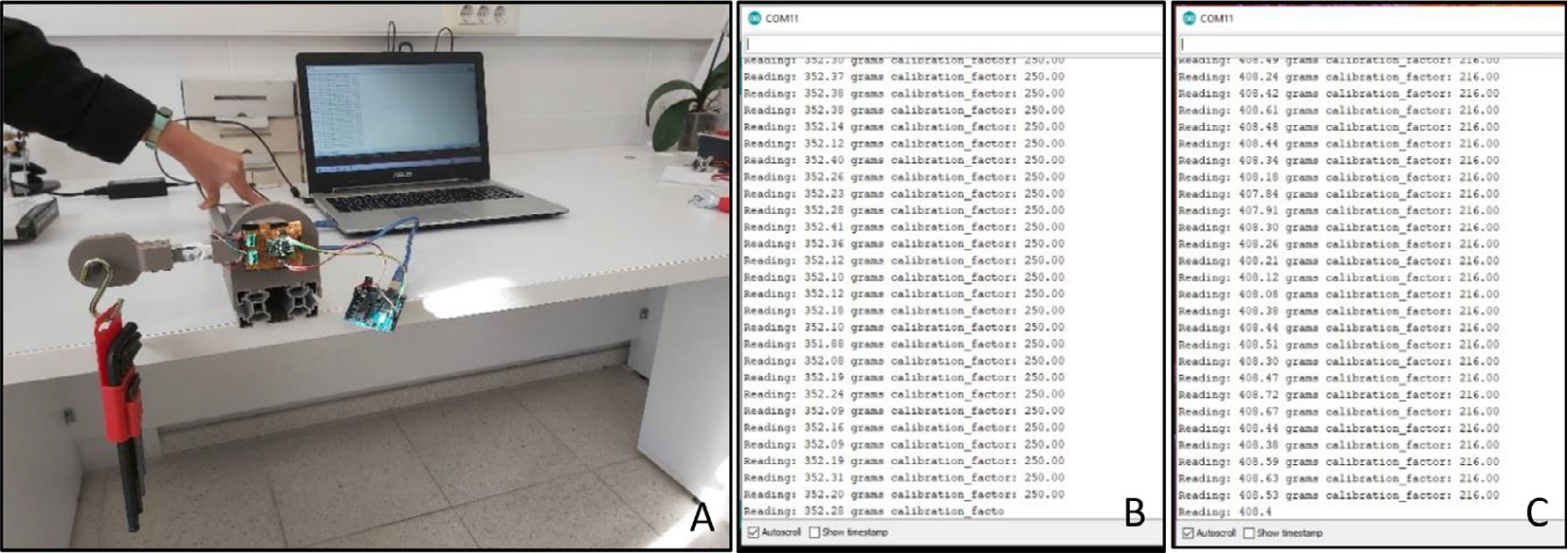
(A) Calibration setup with weights (B) Arduino IDE serial communication monitor with uncorrected calibration factor (C) Arduino IDE serial communication monitor with corrected calibration factor.

After resetting the load cell, system should be placed again as parallel to the ground and known weights must be hanged on the sensed side (Fig. 23A). Using the embedded code on Arduino Uno, the serial reading can be done by using Arduino development environment (Fig. 23B). The variable “calibration_factor” can be changed by sending special characters to the hardware via serial communication (special characters ‘+’ and ‘-‘). By changing this “calibration factor” in the embedded code, it is observed that calculated weight (‘Reading’ in serial communication) changes as well. The correct calibration factor can be determined by the procedure, as it was found to be 216 for the constructed specific system in this study (Fig. 23C). This value should be noted in order to be used in the later processes.

### Velocity control tuning

**Fig. 24:**
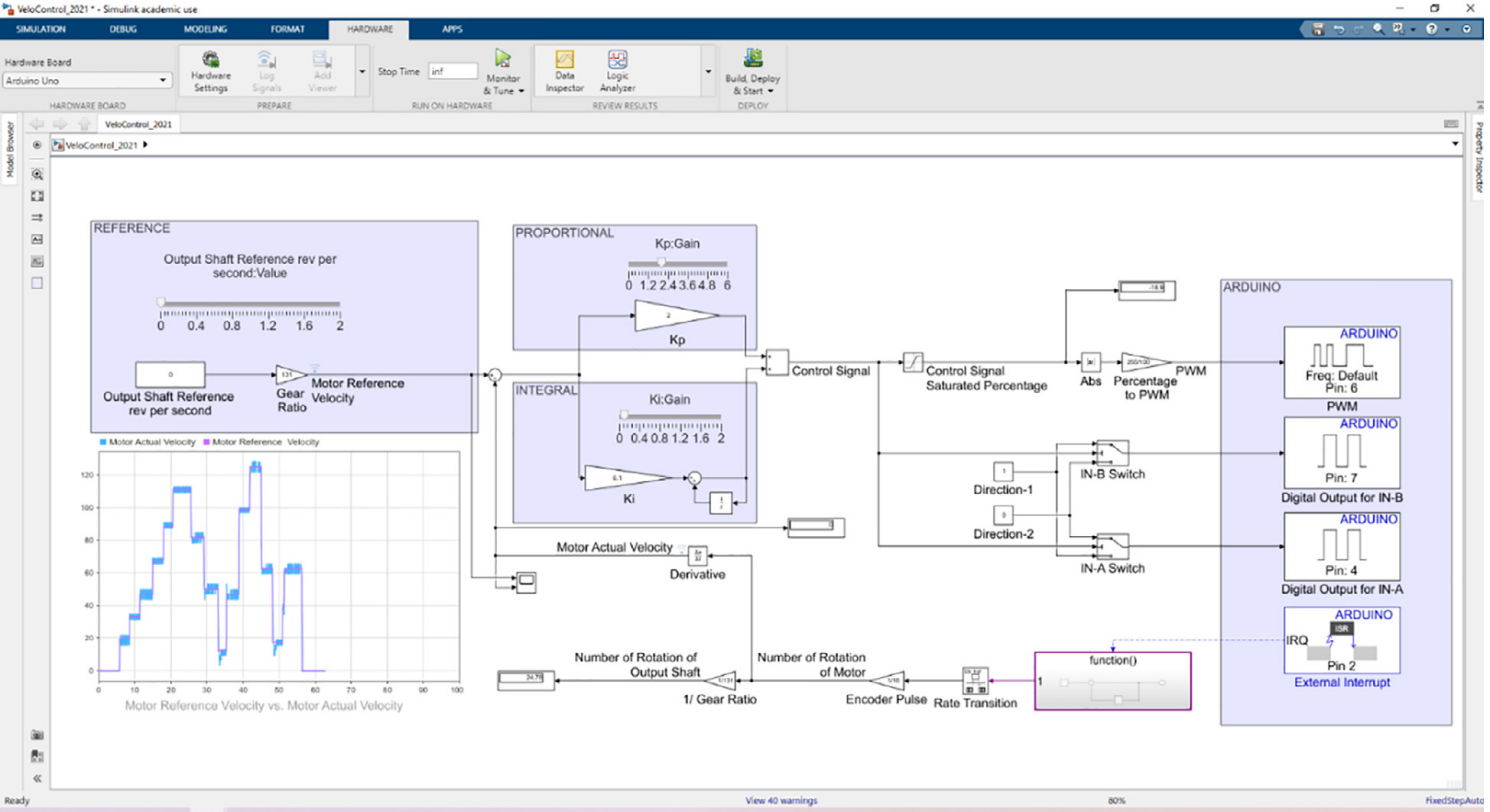
Velocity control tuning simulink model.

Arduino Uno is responsible to control equipped actuator in velocity control mode. In order to use the controller and tune its parameters, Simulink model was created by using “Simulink Support Package for Arduino Hardware”. Control model was constructed in Simulink (Velocity Control Tuning Model.slx in repository) and utilized in external mode, which lead to have real time communication with the hardware (Fig. 24). This helps to have visualization of observed data, and generate easier tuning procedure. In Simulink model for velocity control tuning, velocity reference value of the actuator output shaft, in terms of revolutions per second, can be altered with a slider for values between 0 and 2. As the closed loop needs a feedback in terms of reference unit, velocity of the electric motor must be measured, where built-in actuator encoder was used. Since encoder pulse pins are connected to external interrupt pins of Arduino (pin 2 and pin 3), its pulses were counted with the external interrupt block, which can be found under “Simulink Support Package for Arduino Hardware”. Number of rotations of the actuator extracted by counting the number of total encoder pulses was differentiated in order to get motor shaft velocity. Proportional – Integral (PI) control algorithm was built in the model and the parameters (*K_p_* and *K_i_*) were bind to sliders, which can be adjusted easily. According to the determined control signal value, the direction of the electric motor and the PWM signal was sent to the required hardware pins. PI controller parameters (*K_p_* and *K_i_*) were tuned in real time by altering velocity input reference by the help of inserted sliders in the interface. Changing the parameters, transient and steady state responses were considered and convenient parameters were determined. It is also possible to utilize different tuning methods in the procedure as well.

**Fig. 25:**
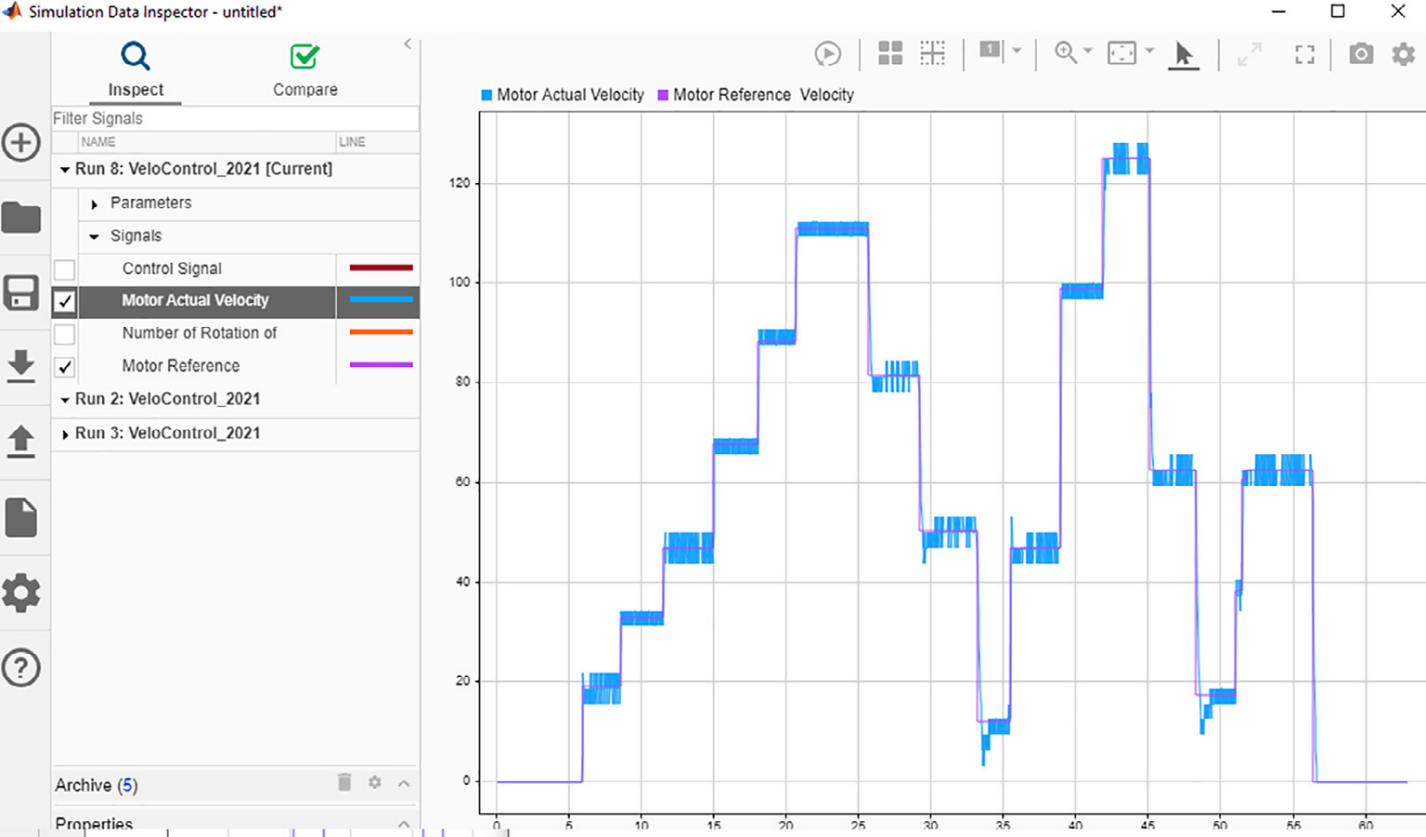
Input-output relation of the tuned system.

In external mode, control performance related variables can be observed and saved in order to be examined later with “Simulink Data Inspector”. For the specific system that was considered in this work, PI control tuning was utilized and input–output relation was determined as in Fig. 25. It was revealed that performance of the system was sufficient when *K_p_* is 2 and *K_i_* is 0.1. These values must be noted so that they can be deployed to the hardware in the rehabilitation usage.

### Velocity control deployment to hardware

**Fig. 26:**
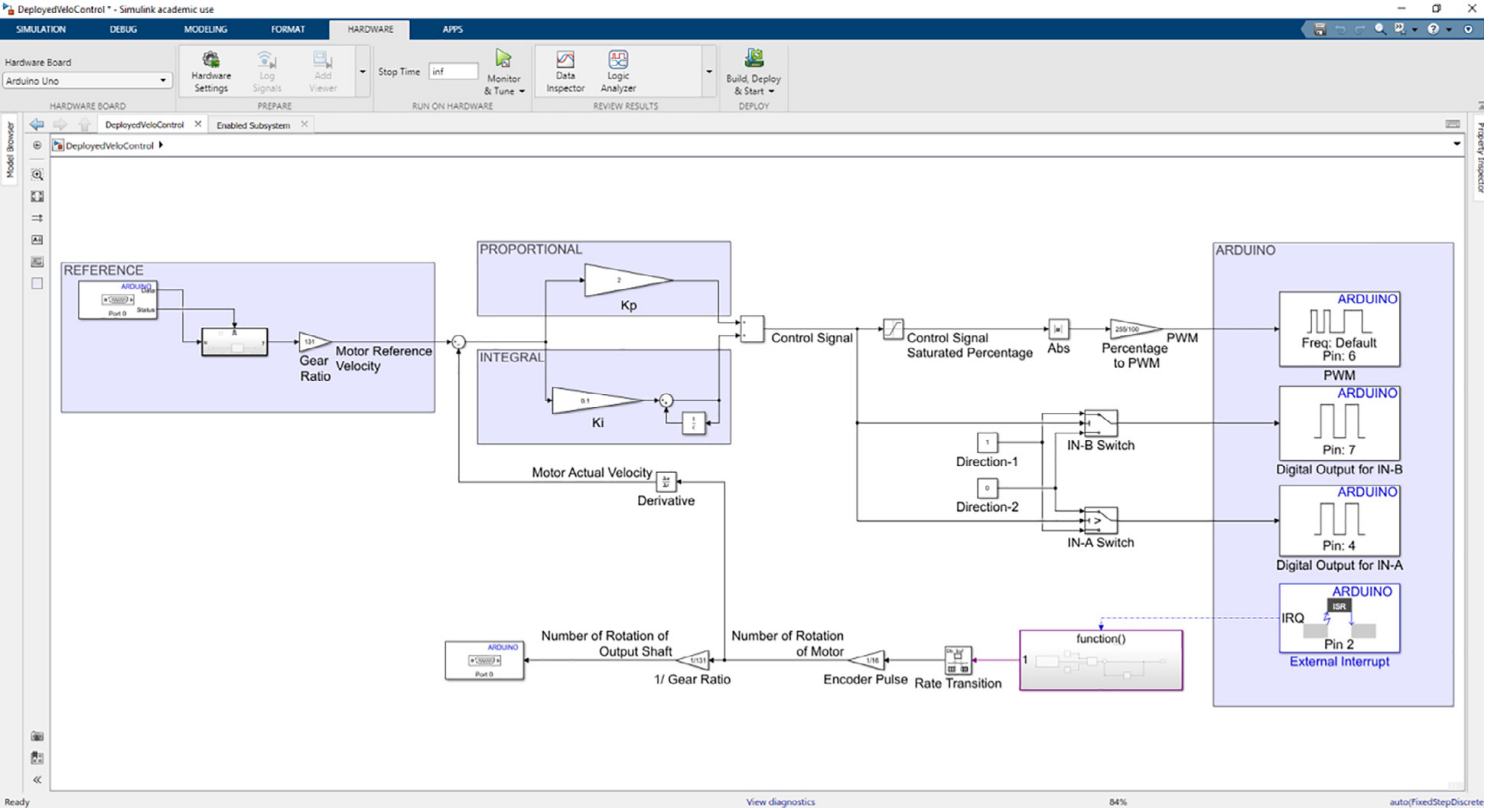
Velocity control deployment to hardware simulink model.

Communication between Arduino Uno and gaming environment that can be executed in any possible operating system is provided by serial communication, thus embedded software in Arduino Uno should have the ability to supply serial communication. In proposed system, Arduino Uno receives the reference velocity of the actuator output shaft in terms of revolutions per second and transmits it via serial communication. Similar to “Velocity Control Tuning Model”, a Simulink model was built and directly deployed to the hardware (Fig. 26).Throughout the procedure, reference input is received by using “Serial Receive” block. After necessary parsing, reference velocity is executed in control system. By the help of encoder pulses, estimated number of rotations is transferred to the operating system with “Serial Transmit” block. Different from the previous tuning model, control parameters are kept constant, in which the tuned and previously noted parameters should be entered.

The proposed model should be deployed to the hardware by the help of “Simulink Support Package for Arduino Hardware”. If Matlab/Simulink intervention is not possible due to any reason, hex file that is prepared from the test variables are also supplied in repository (VelocityControlDeployedModel.hex). It can be downloaded to Arduino by considering that the control parameters were hardcoded to the model with parameters *K_p_* = 2 and *K_i_* = 0.1. The performance with these parameters may not be sufficient for different systems, thus it is recommended to conduct given process.

### Gaming details

Integration of game environment to the system with rehabilitation procedures was carefully handled and all of the explanations in coding were supplied as comments in the study. On the other hand, it should be noted that if it is required to customize given game components for different needs, some critical points must be underlined as an overview. This can lead to understand the power of the software so that customization limits might be much informative for users. Also, thanks to the user interface in gaming environment, communication options and rehabilitation procedure default values are adaptable. Details regarding with the procedural approach, gaming interface and adaptability options will be explained in detail here.

Rehabilitation game and admittance control algorithm was created by using Unity 3D. Gaming environment was built by creating two different Unity scenes; one handles user interface and other handles the game. Algorithms were created by using C# language and game flow was created in a logical way. Admittance control algorithm with its numerical solver was also included in the scripts. Numerical solver for the system was chosen as 4th order Runge-Kutta and its details in admittance control usage were given in Appendix A3. Game level parameters that were utilized in this study were selected to simulate mentioned rehabilitation procedures. Due to the fact that they are only the values to check the performance of the proposed system, during real rehabilitation treatment, they should be altered by consulting medical experts on the field.

**Fig. 27:**
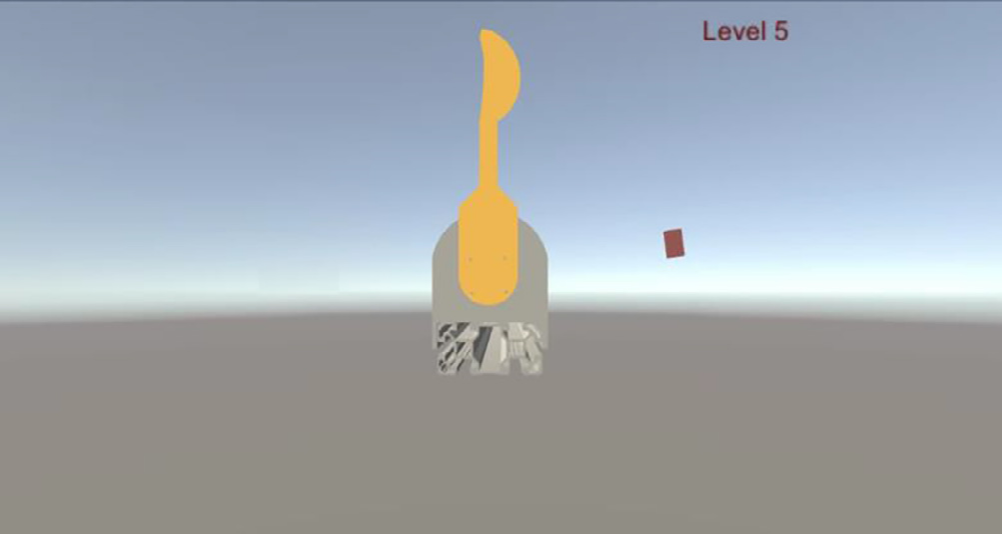
Rehabilitation game connected to PARS.

The main concept behind the developed game for this study is in fact simple. During the game the main goal of the player is to be able to catch a virtual object that continuously rotates around the Swatter (Fig. 27) that is driven with the “Force Link” of the PARS. Interaction between the user and the handle is utilized with the admittance control algorithm that is embedded in gaming scripts. Rehabilitation procedures are included as patient passive, patient active with assistance and patient active with resistance modes.

In the levels where the patient is active, interaction control is included and rotational velocity of the virtual object is bind to the velocity of the user handle, which is the velocity of the swatter. Therefore, in order to catch the object, user should move robot link as fast as possible. If the motion is interrupted and stopped by the user for some reason, virtual object rotates with a predefined minimum velocity and crashes to the swatter to render the level as failed. Once the virtual object is caught with the swatter in the rotational direction, dedicated level is counted as achieved.

Default levels of the game were shaped by considering rehabilitation modes. First, the system aims to create plasticity in the brain, where there exist patient-passive motions. Here, the control structure is constructed basically with a velocity controller, where there is no need for an applied interaction from the user. Later, the levels with patient-active modes come into action. In such levels, saturation force, virtual admittance coefficients and object rotational velocity dependency define level difficulties. These coefficients can be edited by users. In active assistance cases, user forces are doubled until *f_saturation_* is reached, in which assistive force is very dominant. If the *f_saturation_* is zero, then the motion is directly affected by the virtual admittance coefficients. In the active resistance cases, in order to begin the movement, user should apply a force bigger than *f_saturation_*.

In order to keep the game and interaction control behind the procedure adaptable to real treatment scenarios under the supervision of medical experts, all the necessary options that might differ from user to user were designed to be editable. Therefore, a configuration file (config.json) that keeps information about communication, default game levels, edited game levels, user names, save folder etc. was prepared. Configuration file can be edited via user interface as well, however if it is needed to have a deeper change in game logic, this file should be examined. In case the game is required to be updated, all the necessary information comments were provided in C# scripts.

### Pre-arrangements

In order to start using PARS as a rehabilitation system with game interface, required procedures that were also mentioned before should be conducted for an efficient operation. These procedures can be simply summarized as below,

• Calibration of the load cell
• Tuning velocity control parameters
• Deploying velocity control model to Arduino Uno

**Fig. 28:**
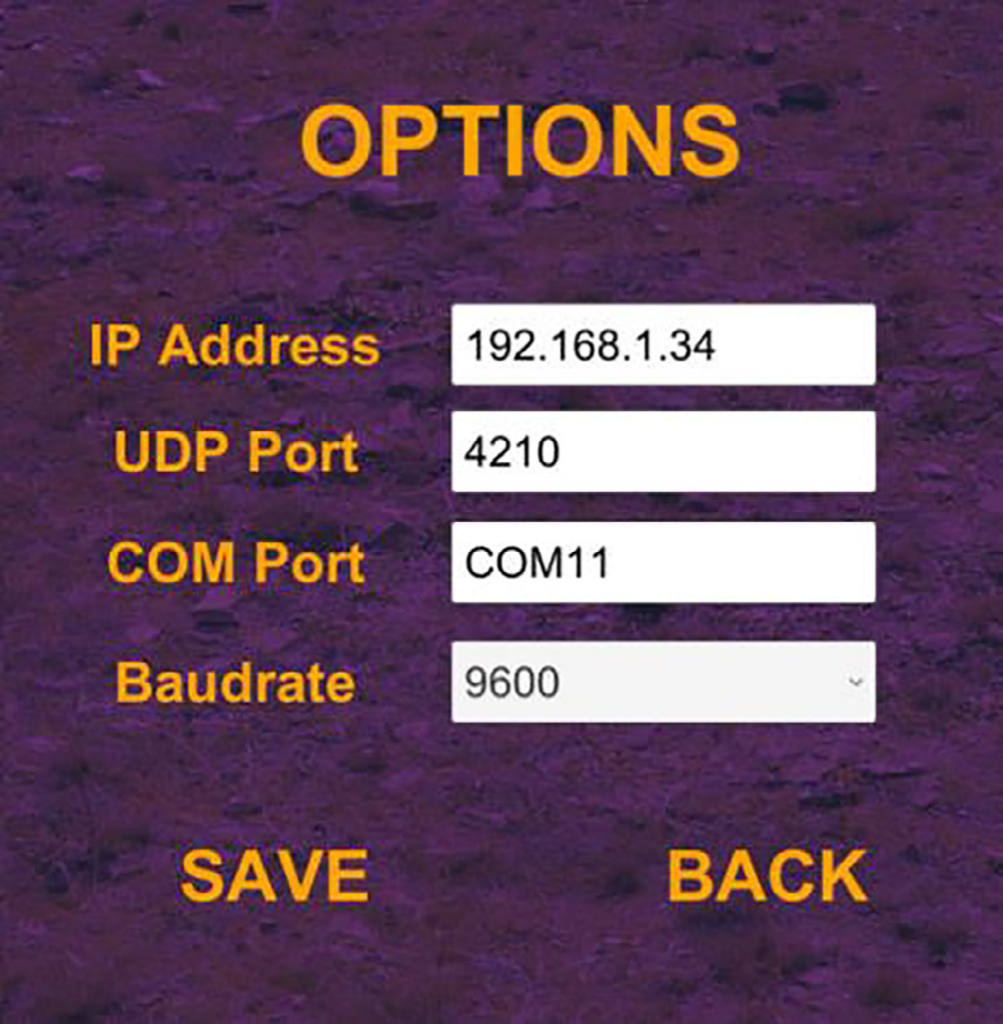
Options page of game.

As a next step, PARS should be adapted in order to be utilized in an operating system, in order to have proper communication via Wi-Fi with UDP and via serial communication. Operating system and ESP8266-01 should be connected to a common wireless network for Wi-Fi connection. Communication in UDP is possible through a predefined port and ip-address, therefore chosen port must be the same with the one in ESP8266-01 embedded code. Also the ip-address given to ESP should be known and inserted in game options (Fig. 28). Serial communication options can also be changed from the options in game (Fig. 28). Serial communication port with Arduino Uno and operating system can also be specified as well as the baudrate, which should be the same as in specified “Hardware Implementation” tab in “Velocity Control Deployed Model” (its default is 9600).

### Overall operation

**Fig. 29:**
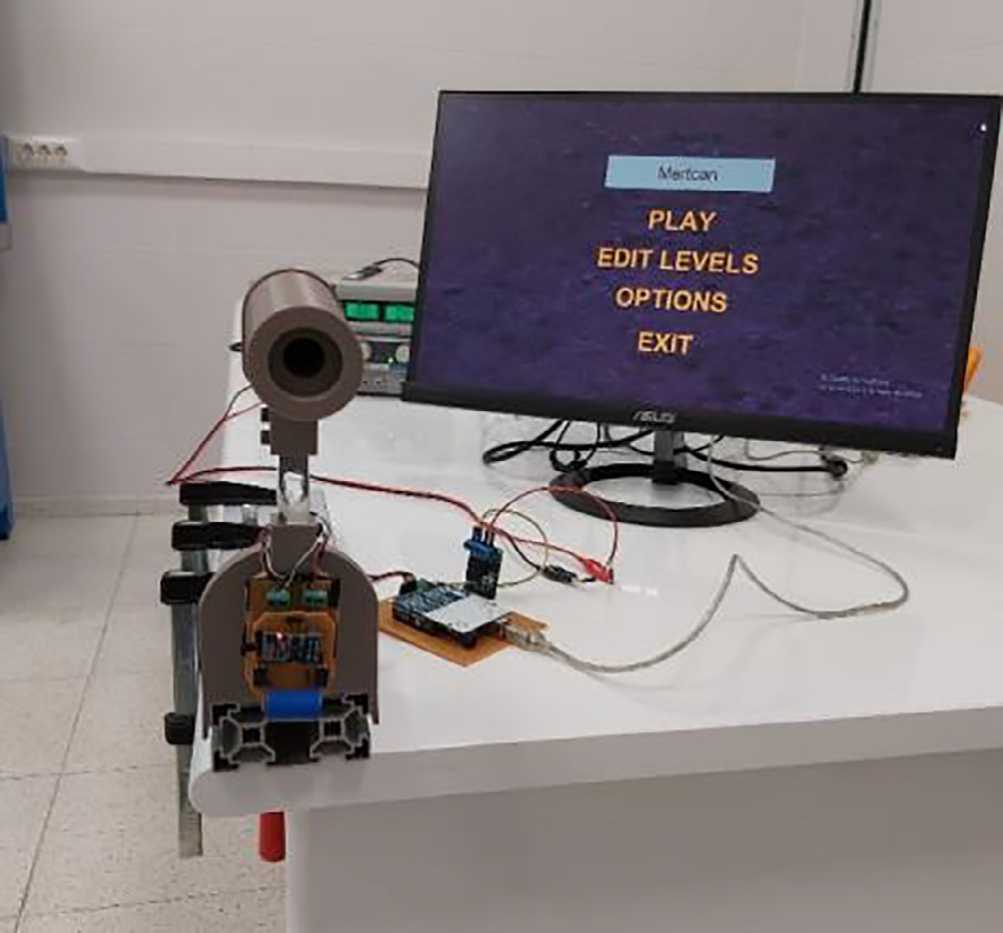
Initial position of PARS before turning on power switch.

During the operation, operating system should be connected to Arduino Uno via the USB, motor driver should be powered on (12 V for this configuration) and the battery on “Wireless Communication Circuit” should be placed to its headers. Before turning on the power switch of “Wireless Communication Circuit”, PARS should be taken to vertical position as in Fig. 29 and be kept in this position for a few seconds, in order to have a zero load on the load cell during the initialization.

**Fig. 30:**
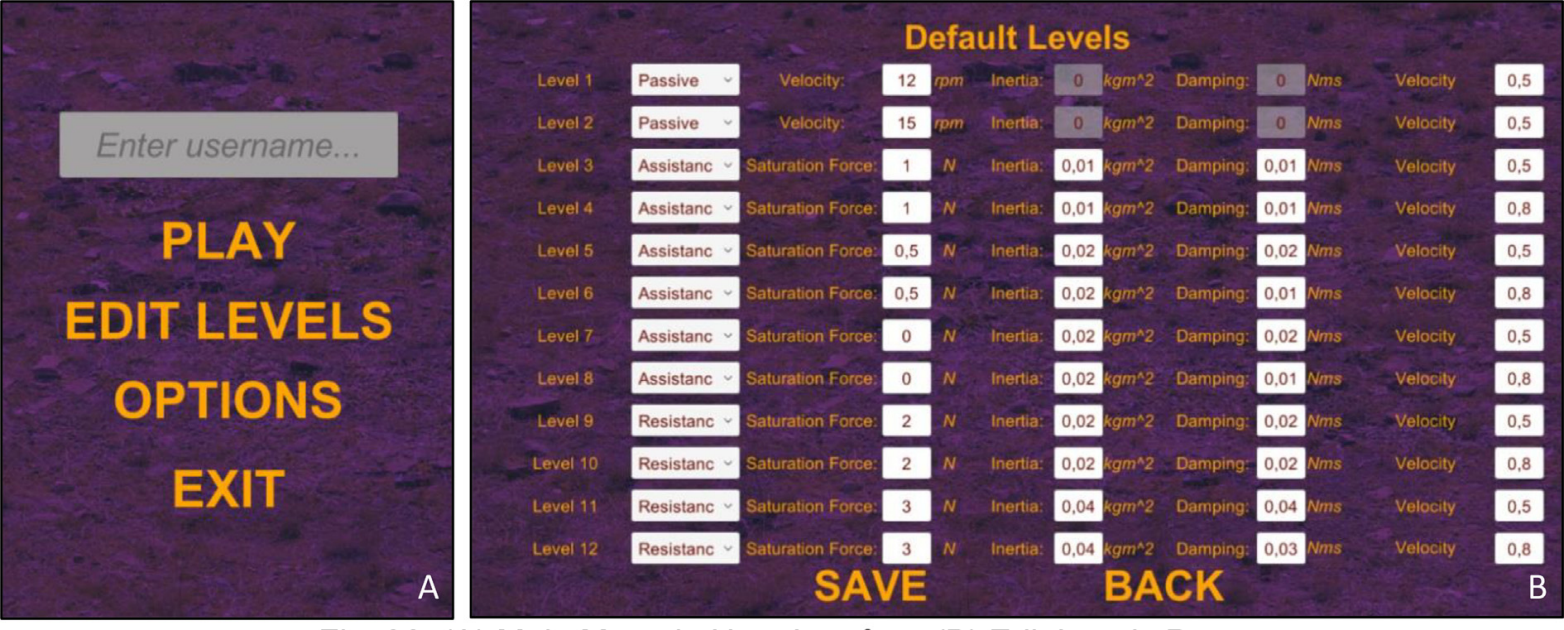
(A) Main menu in user interface (B) Edit levels page.

Following the initialization of the hardware, supplied game can be executed as ready for necessary arrangements to be made. “Main Menu” of the user interface in game starts as shown in Fig. 30A. Level parameters for rehabilitation control (assistance, resistance, saturation force, virtual rotational inertia, virtual damping coefficient, object velocity multiplier) can be edited in “Edit Levels” selection (Fig. 30B). By default, first and second levels are reserved for passive rehabilitation, where there exist no interaction between the user and the handle. It is just a simple velocity control where the reference value can be altered from the page. In such levels, user should hold the handle and the system executes the movement without considering any external affects, which aims to create plasticity in the brain. Default level parameters are defined as in Fig. 30B, however this predefined default values can be changed via configuration file (config.json). After changing the parameters, it is always possible to click on “Default Levels” button in order to undo altered parameters.

**Fig. 31:**
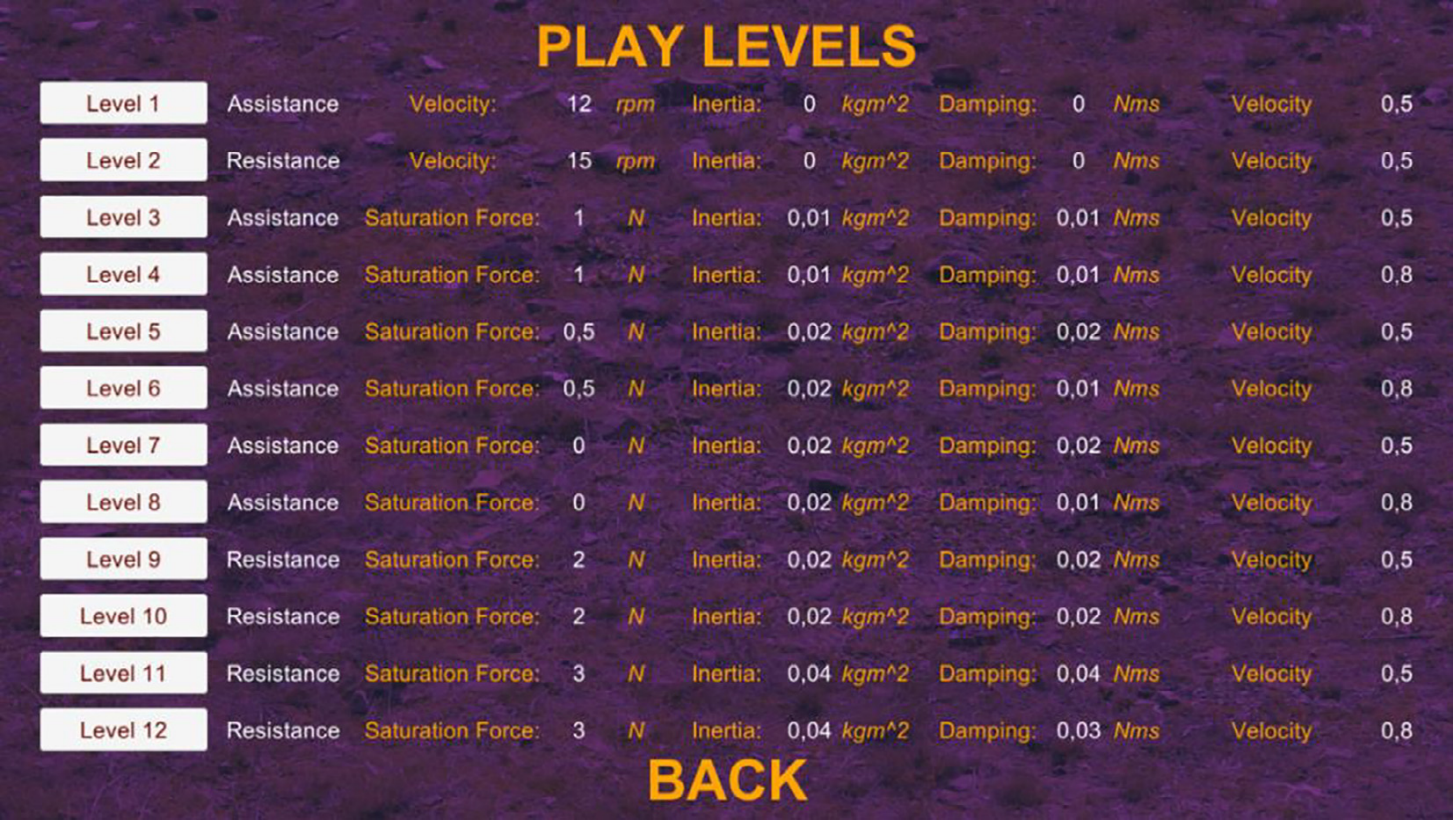
Play levels page.

Prior to the start, username should be entered in input text field in order to identify the patient. After all the configurations are utilized with respect to the needs, “Play” button can be clicked. Parameters about levels are visualized in opened page, in which all levels can be chosen by clicking specified level buttons (Fig. 31).

**Fig. 32:**
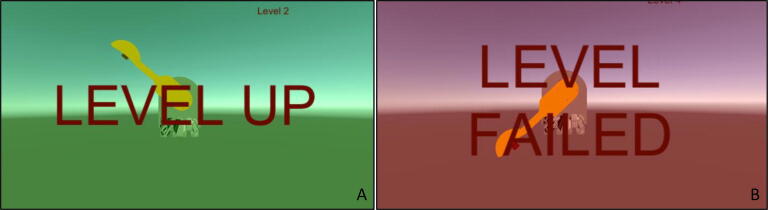
(A) Level is achieved (B) Level is failed.

After the game is started, control algorithm is executed with specified parameters. The swatter is moved by disturbing the handle of the PARS in real life, unless it is in passive mode. If the rotating virtual object is caught by the swatter, the level will be achieved and positive visual feedback is given to the user (Fig. 32A). If the virtual object rotates faster than the swatter and crashes behind, the level will be failed (Fig. 32B). In both cases, saving the extracted data becomes optional. If it is requested to save the data, it is needed to click “Save” button, otherwise “Close” button. The data is saved under the built folder “UpperArmRehabGame\UpperArmRehabGame_Data” with the extension of csv and named as “username_year_month_day_hour_minutes_seconds.csv”.

It should be noted that if the Unity codes or structure is desired to be edited for any reason, the system must be built again in order to prepare an executable file. For proper implementation, config file (config.json) must be relocated under the folder “UpperArmRehabGame_Data”, which was created after building. All saved data can be visualized and post processed by using appropriate software. Game can be terminated by clicking to “Exit” button in the main menu (Fig. 30A). It should be noted that the battery used in the system is a rechargeable battery, therefore it should be recharged properly. Since it is a 3.6 V battery, the voltage given must not be exceeded 4.2 V during charging process.

## Validation and characterization

In order to validate proposed system, one healthy adult played designed rehabilitation game with the default difficulty levels given in Fig. 30B. Initial position of the swatter was 90 ° with respect to the rotating virtual object. Results in Table 1 show that player increases the effort with respect to the changes in difficulty parameters. Since level 1 and level 2 were reserved for passive rehabilitation, they were not included in tabulated results. There is an undeniable correlation between the admittance coefficients and the applied force as well as energy spent. For example, by keeping the saturation force and the rotational velocity multiplier unchanged, the effect of the admittance control coefficients can be verified in level 3 and level 5 or level 4 and level 6. Saturation force affects the overall energy and intention force less, but still can be very useful in applied algorithms, especially its initial support to move the system is vital for active assistance cases. Also, in resistance cases, user must apply more force to initiate the motion, together with the effect of admittance dynamics. The most energy consumed levels are the ones with high velocity ratio. The reason behind this is the fact that it is harder to catch the rotating object and it takes time for it to be caught.

**Table 1:** Validation results of the game placed by a healthy adult.

**Levels**	**Average Velocity [°/s]**	**Average User Force [N]**	**Rotation [°]**	**Estimated Energy [N rad]**	**Duration [s]**
Level 3	8.19	6.08	565	59.93	3.75
Level 4	8.42	6.18	1190	128.29	9.05
Level 5	6.08	9.83	583	99.97	5.25
Level 6	7.02	11.41	1433	285.23	10.60
Level 7	7.43	12.87	580	130.22	4.25
Level 8	8.07	13.68	1397	333.38	8.95
Level 9	7.20	14.30	569	141.94	4.25
Level 10	7.52	14.85	1422	368.37	9.85
Level 11	4.62	18.55	587	189.95	6.75
Level 12	4.87	19.45	1465	497.07	15.45

## Conclusion

This study successfully proposed a compact single degree of freedom robotic system that can be utilized for rehabilitation treatments. The system was integrated with a designed virtual game environment in order to take advantage of cognitive effects. All of the required design files were provided to create an open source hardware platform that can be enhanced by the researches working in the field. Compared with the authors’ previous work [34], the system was advanced to the next step. Electric motor was replaced with a basic one that cost dramatically less and available for the majority so that reproducibility of the system can increase. Furthermore, outer loop in admittance control was bind to the developed game software so that proper game flow was provided. All difficulty levels can be adaptable according to the requirements and can be customized for the user by the host so that the game software handles all the necessary utilization in the control algorithm. In the end, user friendly design makes it possible to be integrated without the necessity of strong technical background. With the possibility of game software adaptation to be operable in Android or IOS environment, gaming environment can be advanced to different platforms. The effect of the custom games in neuro-rehabilitation can also be examined deeply with the proposed system. Since the promising results were acquired with the implementation of stable game flows, the clinical trials can be utilized hereafter. On the other hand authors strongly advise that during real rehabilitation treatment procedures on actual patients, gaming parameters should be altered by consulting medical experts on the field.

### CRediT authorship contribution statement

**Mertcan Koçak:** Conceptualization, Methodology, Investigation, Validation, Writing – original draft, Software, Visualization, Data curation. **Erkin Gezgin:** Conceptualization, Methodology, Writing – original draft, Supervision, Project administration.

## Declaration of Competing Interest

The authors declare that they have no known competing financial interests or personal relationships that could have appeared to influence the work reported in this paper.
