# Supplementary material for: PARS, low-cost portable rehabilitation system for upper arm
Source: HardwareX. 2022 Mar 23;11:e00299. doi: 10.1016/j.ohx.2022.e00299 (PMC9058851; doi:10.1016/j.ohx.2022.e00299)
Supplement: Supplementary data 3 [file mmc3.docx]

**Appendix A1. Load Cell Calibration Software**

The code for calibration of load cell is adapted from Nathan Seidle’s “SparkFun_HX711_Calibration.ino” with the help of bodge’s “HX711.h” library, which is released under a GNU General Public License.

**Calib.ino;**

/* Calibration procedure of load cell used in the project.

This sketch is originally built by Nathan Seidle of SparkFun Electronics

with the help of contributors of "HX711.h" library and adapted for this project.

bogde's library ("HX711.h") is released under a GNU GENERAL PUBLIC LICENSE

HX711.h Library: https://github.com/bogde/HX711

Original Sketch: https://github.com/sparkfun/HX711-Load-Cell-Amplifier/blob/master/firmware/SparkFun_HX711_Calibration/SparkFun_HX711_Calibration.ino

Arduino pin 2 -> HX711 CLK

Arduino pin 3 -> HX711 DOUT

Arduino pin 5V -> HX711 VCC

Arduino pin GND -> HX711 GND

*/

#include "HX711.h" // Including library

#define DOUT 2 // DOUT pin

#define CLK 3 // CLK pin

HX711 scale;

float calibration_factor = 216; // calibration factor to be adjusted

float units;

float ounces;

void setup() {

Serial.begin(9600); // Starting serial communication

// Press + to increase calibration factor

// Press - to decrease calibration factor

scale.begin(DOUT, CLK); // Beginning of scale according to defined pins

scale.set_scale();

scale.tare(); //Reset the scale to 0

long zero_factor = scale.read_average(); // Get a baseline reading

}

void loop() {

scale.set_scale(calibration_factor); // Adjust to updated calibration factor

Serial.print("Reading: ");

units = scale.get_units(), 10;

if (units < 0) {

units = 0.00;

}

ounces = units * 0.035274;

// Readings according to grams, it can be changed to ounces.

Serial.print(units);

Serial.print(" grams");

Serial.print(" calibration_factor: ");

Serial.print(calibration_factor);

Serial.println();

// Adjusting calibration factor via serial communication

if(Serial.available())

{

char temp = Serial.read();

if(temp == '+')

calibration_factor += 1;

else if(temp == '-')

calibration_factor -= 1;

}

}

**Appendix A2. ESP8266-01 UDP Wi-Fi Software**

The code for ESP8266-01 connection with UDP and reading load cell is adapted from bodge’s “HX711.h” library, which is released under a GNU General Public License.

**Esp_udp.ino;**

/* This code is created with ESP8266WiFi.h - esp8266 Wifi support that is based on WiFi.h from Arduino WiFi shield library,

that was modified by Ivan Grokhotkov, December 2014 and WiFiUdp.h - Library for Arduino Wifi shield.

bogde's library ("HX711.h") is released under a GNU GENERAL PUBLIC LICENSE and is used with ESP8266-01

HX711.h Library: https://github.com/bogde/HX711

ESP8266-01 pin 5 (GPIO 0) -> HX711 CLK

ESP8266-01 pin 3 (GPIO 2) -> DOUT

*/

#include <ESP8266WiFi.h>

#include <WiFiUdp.h>

#include "HX711.h"

// ESP8266 Definitions

#define ARCH_ESPRESSIF ARDUINO_ARCH_ESP8266

#define FAST_CPU

#define CLK 0

#define DOUT 2

// WiFi Definitions

#define WIFI_SSID "SSID" // SSID of the LAN Network

#define WIFI_PASS "PASSWORD" // Password of the LAN Network

#define UDP_PORT 4210 // UDP Port (it should be same as the one specified in Game Options Menu)

HX711 scale;

float calibration_factor = 216; // Determined value in calibration procedure

int units;

// UDP

WiFiUDP UDP;

char packet[255]; // Receive UDP packet

uint8_t udpTxArray[3]; // Send UDP Packet

bool isRecieved = false; // Receive logic boolean

void setup() {

// Setup serial port

Serial.begin(9600);

delay(5000);

Serial.println();

// Begin WiFi

WiFi.begin(WIFI_SSID, WIFI_PASS);

// Connecting to WiFi...

Serial.print("Connecting to ");

Serial.print(WIFI_SSID);

// Loop continuously while WiFi is not connected

while (WiFi.status() != WL_CONNECTED)

{

delay(100);

Serial.print(".");

}

// Connected to WiFi

Serial.println();

Serial.print("Connected! IP address: ");

Serial.println(WiFi.localIP());

// Begin listening to UDP port

UDP.begin(UDP_PORT);

Serial.print("Listening on UDP port ");

Serial.println(UDP_PORT);

Serial.println("Load Cell with ESP8266");

Serial.println("...");

delay(1000);

Serial.println("scale begin...");

// LOAD CELL SETTINGS

scale.begin(DOUT, CLK);

scale.set_scale(calibration_factor); //Adjusted calibration factor

scale.tare(); //Reset the scale to 0

// No touch to load cell at start

long zero_factor = scale.read_average(); //Get a baseline reading

Serial.println("read average...");

}

void loop() {

// Do nothing until receive data via UDP

// IP address of the operating system is determined automatically, when the data is received

// In Unity game software, UDP message is sent at the beginning of the connection

while (!isRecieved)

{

int packetSize = UDP.parsePacket(); // Receive UDP packet

if (packetSize) { // If packet available

int len = UDP.read(packet, 255);

if (len > 0)

{

packet[len] = '\0';

}

isRecieved = true;

break;

}

}

// Load Cell Reading

int loadCellKg = ReadLoadCell();

// Definition for positiveness is added at the beginning of UDP packet (1 for positive, 0 for negative)

int isPositive = 1;

if (loadCellKg < 0) {

isPositive = 0;

loadCellKg = -1*loadCellKg;

}

// If the connection is lost, local port of operating system that drives game changes.

// Therefore, UDP listens its port if there exists any receiving package.

int packetSize = UDP.parsePacket(); // Receive UDP packet

if (packetSize) { // If packet available

int len = UDP.read(packet, 255);

if (len > 0)

{

packet[len] = '\0';

}

}

else // if there is no receiving packet, then the data is sent via UDP to host

{

// UDP Sending Array Creating

uint8_t udpTxArray[3] = { highByte(loadCellKg), lowByte(loadCellKg), lowByte(isPositive)};

// Send packet to first receiving remoteIP over remotePort

UDP.beginPacket(UDP.remoteIP(), UDP.remotePort());

UDP.write(udpTxArray,sizeof(udpTxArray));

UDP.endPacket();

}

}

// Reading load cell function

int ReadLoadCell()

{

units = (int)(scale.get_units());

return units;

}

**Appendix A3. 4^th^ Order Runge Kutta Numeric Solver Implementation**

Admittance control defines the dynamic behaviour between the input torque and the output rotational velocity, with specified virtual environment. The utilized differential equation for this purpose is given in Equation 3.

|  | $\frac{d\omega}{dt}=\frac{\tau-b_{v}\omega}{J_{v}}$ | (3) |
| --- | --- | --- |

where $\omega$ is the rotational velocity in terms of *rad/s* (radian per second), $\tau$ is the input torque, $b_{v}$ is the virtual damping coefficient in terms of *Nms* (Newton-meter-seconds) and $J_{v}$ is the virtual rotational inertia in terms of *kgm^2^*.

The specified differential equation can be determined with different solvers numerically, in this case it is chosen to be solved with 4^th^ order Runge Kutta method due to its easiness in implementation (Equation 4).

|  | $\frac{d\omega}{dt}=f\left( t,w \right)$  $\omega_{n+1}=\omega_{n}+\frac{1}{6}h\left( k_{1}+{2k}_{2}+{2k}_{3}+k_{4} \right),$  $t_{n+1}=t_{n}+h$ | (4) |
| --- | --- | --- |

where, $k_{1}=f(t_{n},\omega_{n})$, $k_{2}=f(t_{n}+\frac{h}{2},\omega_{n}+h\frac{k_{1}}{2})$, $k_{3}=f(t_{n}+\frac{h}{2},\omega_{n}+h\frac{k_{2}}{2})$, $k_{4}=f(t_{n}+h,\omega_{n}+hk_{3})$ and $h$ is the time step. Implementation of the numerical method to solve the Equation 3 in gaming environment is utilized with the class structure “AdmittanceControl.cs” that is built with C#.

**AdmittanceControl.cs;**

using UnityEngine;

public class AdmittanceControl : MonoBehaviour

{

// No need to fill constructor of the class.

public AdmittanceControl()

{

}

// ControlOut with required parameters that returns reference rotational velocity of the output shaft in terms of rev/s.

public double ControlOut(double torque, float dt,double w0, double virtualDamping, double virtualInertia)

{

return rungeKutta(torque,dt,w0,virtualDamping,virtualInertia);

}

// Differential equation to solve with numerical method

static double dwdt(double t, double w,double c, double J)

{

return ((t-c*w) / J);

}

// 4th order Runge-Kutta method implementation

static double rungeKutta(double t, double dt, double w0, double c, double m)

{

// Step size or step height dt

double k1, k2, k3, k4;

double w1 = w0;

// Apply Runge-Kutta Formulas to find next value of rotational velocity (w1)

k1 = dwdt(t, w0,c,m);

k2 = dwdt(t+0.5*dt, w0+0.5*dt*k1,c,m);

k3 = dwdt(t+0.5*dt, w0+0.5*dt*k2,c,m);

k4 = dwdt(t+dt, w0+dt*k3,c,m);

// Update next value of w

w1 = w1 + (1.0/6.0)*(k1 + 2*k2 + 2*k3 + k4)*dt;

return w1;

}

}
