# Supplementary material for: PARS, low-cost portable rehabilitation system for upper arm
Source: HardwareX. 2022 Mar 23;11:e00299. doi: 10.1016/j.ohx.2022.e00299 (PMC9058851; doi:10.1016/j.ohx.2022.e00299)
Supplement: Supplementary data 4 [file mmc4.zip › Design Files.docx]

Design Files were uploaded to the system as a *.rar file under the ‘design files’ upload section with ‘3DModels’ item name
